# Soft cell-derived hybrid microparticles with platelet decoys for enhanced cancer chemotherapy

**DOI:** 10.1016/j.apsb.2026.03.028

**Published:** 2026-03-19

**Authors:** Nana Bie, Shiyu Li, Kaili Sun, Jianye Li, Xin Li, Xiaojuan Zhang, Muzi Tian, Zixiang Xie, Yixi Xiao, Yujie Zhang, Zixi Wang, Yizhou Huang, Yinmei Zhu, Xiangliang Yang, Lu Gan, Tuying Yong

**Affiliations:** a National Engineering Research Center for Nanomedicine, College of Life Science and Technology, Huazhong University of Science and Technology, Wuhan 430074, China; b Key Laboratory of Molecular Biophysics of the Ministry of Education, College of Life Science and Technology, Huazhong University of Science and Technology, Wuhan 430074, China; c Hubei Key Laboratory of Bioinorganic Chemistry and Materia Medica, Huazhong University of Science and Technology, Wuhan 430074, China

**Keywords:** Tumor targeting delivery, Extracellular vesicles, Platelet decoys, Platelet-tumor interaction, Softness, Post-surgery anti-tumor therapy, Tumor metastasis, Toll-like receptor-4

## Abstract

Platelets play a critical role in tumor development, metastasis and chemoresistance, making the effective killing of tumor cells and simultaneously targeted disruption of platelet functions essential for improving cancer treatment outcomes, especially in post-surgical malignant tumor patients. Here, we develop soft hybrid microparticles (3D-PMPs) by fusing tumor-repopulating cell-derived microparticles with inactivated platelet membranes to deliver the anticancer agent doxorubicin (DOX@3D-PMPs). Leveraging their unique softness, DOX@3D-PMPs demonstrate superior tumor accumulation, deep tumor penetration, and enhanced internalization into tumor cells, leading to efficient tumor cell killing. Additionally, 3D-PMPs function as highly targeted platelet decoys to disrupt platelet-tumor cell interaction and reduce platelet-driven tumor proliferation and metastasis. Mechanistically, Toll-like receptor 4 (TLR-4) presented on 3D-PMPs is responsible for their platelet decoy function. DOX@3D-PMPs demonstrate significantly enhanced therapeutic efficacy in both orthotopic 4T1 breast tumors and post-surgical orthotopic 4T1 breast tumors. This work offers a novel and effective approach to enhance the therapeutic outcomes in cancer treatment, particularly in post-surgical settings.

## Graphical abstract

Soft DOX-loaded hybrid platelet decoys enhance tumor accumulation, penetration, and cellular uptake, while blocking platelet-tumor interactions *via* TLR-4 to suppress metastasis and potentiate chemotherapy.

**Figure:**
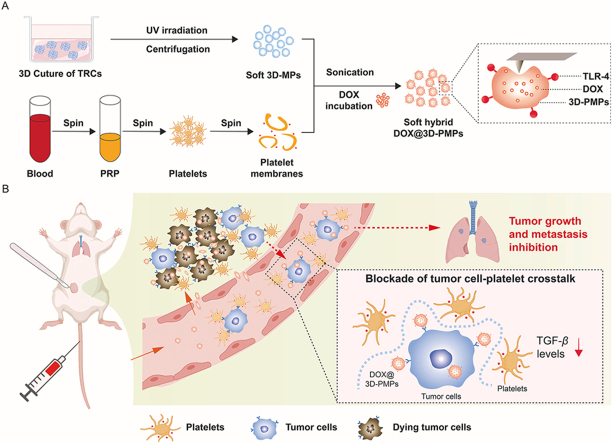

## 1 Introduction

Tumor metastasis is the leading cause of cancer-related deaths. Although surgery is a standard treatment for most malignant solid tumors, it is often followed by neoadjuvant chemotherapy and immunotherapy to eliminate residual tumor cells and prevent metastasis^1^^,^^2^. However, the prognosis for many patients, especially those with highly aggressive tumors, remains poor. A key obstacle to treatment success is the complex tumor microenvironment, which consists of diverse cell types, extracellular matrix components, and cytokines that promote tumor growth, invasion, and resistance to therapy^3^, ^4^, ^5^. Therefore, developing comprehensive strategies that effectively kill tumor cells and simultaneously target the remodeling of the tumor microenvironment is crucial for improving therapeutic outcomes.

Platelets, primarily known for their role in coagulation, have recently gained significant attention for their involvement in promoting tumor growth and metastasis, particularly in patients undergoing surgical excision, where platelet recruitment to the resected tumor site can exacerbate metastatic processes^6^, ^7^, ^8^. Platelets contribute to metastasis by adhering to tumor cells and releasing transforming growth factor-*β* (TGF-*β*), which drives the epithelial–mesenchymal-like transition of tumor cells and enhances their invasiveness^9^. Additionally, platelets facilitate the adhesion of tumor cells to vessel walls, aiding their extravasation and subsequent entry into the circulation. Once in circulation, platelets form a protective thrombus around tumor cells, shielding them from shear stress and immune surveillance to promote their successful colonization at distant metastatic sites^10^, ^11^, ^12^, ^13^, ^14^. Furthermore, platelets have been shown to protect tumor cells from chemotherapeutic drug cytotoxicity by decreasing drug uptake^11^ and reducing sensitivity through microRNA transfer^13^. Consequently, targeting platelet-tumor interactions has emerged as a promising strategy to inhibit tumor growth and metastasis, thereby enhancing therapeutic efficacy. Current approaches, including the use of anti-platelet agents such as nitric oxide^15^, ticagrelor^16^^,^^17^, and specific antibodies like R300 delivered *via* nanoparticles^18^^,^^19^, have demonstrated potential in impairing tumor-specific platelet functions and reducing platelet-induced tumor growth and metastasis. However, these strategies are often limited by substantial bleeding risks due to systemic platelet inhibition, poor tumor specificity, and narrow therapeutic windows. Moreover, antibody- or drug-based platelet blockade typically requires complex manufacturing and still exhibits modest efficacy *in vivo*, collectively restricting their clinical translation. These limitations underscore the urgent need for safer and more tumor-specific approaches, which can locally disrupt platelet-tumor interactions without compromising systemic hemostasis.

Platelet decoys represent an innovative anti-platelet therapeutic strategy that mimics the natural surface of platelets to capture and neutralize circulating tumor cells^20^^,^^21^. By specifically inhibiting the interaction between platelets and tumor cells, platelet decoys provide a highly selective approach to inhibit tumor growth and metastasis, potentially reducing the systemic side effects associated with conventional anti-platelet therapies^22^^,^^23^. Despite their advantages, the clinical application of platelet decoys encounters considerable hurdles, primarily due to the need for effective targeted delivery to tumor tissues to maximize their therapeutic efficacy. Extracellular vesicles, including microparticles (MPs) and exosomes, have received significant attention as drug delivery carriers due to their inherent targeting properties, excellent biocompatibility, and ease of modification^24^, ^25^, ^26^, ^27^. In our previous work, we developed tumor-repopulating cell (TRC)-derived MPs (3D-MPs), which demonstrated unique softness and deformability to promote tumor accumulation, deep tumor penetration and efficient cellular uptake of drugs by tumor cells^28^^,^^29^. Combining 3D-MPs with platelet decoys might offer a synergistic approach to enhance the inhibition of tumor growth and metastasis, ultimately enhancing the efficacy of cancer treatment.

**Figure 1:**
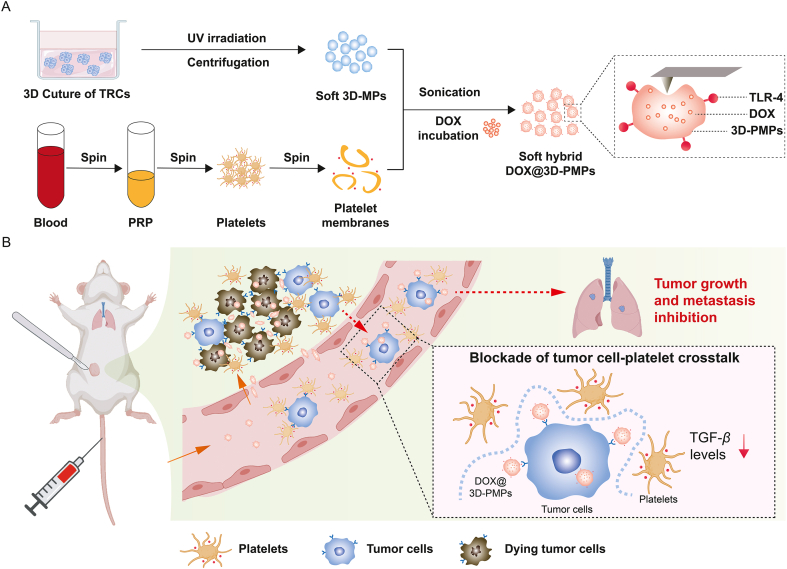
Schematic of DOX@3D-PMPs for enhanced cancer chemotherapeutic efficacy. (A) Schematic illustration of the preparation of DOX@3D-PMPs. DOX@3D-PMPs were obtained by loading DOX to 3D-MPs fused with the fragmented platelet membranes using sonication at a protein ratio of 3:1. (B) Schematic illustration of DOX@3D-PMPs as platelet decoys for enhanced cancer chemotherapy. DOX@3D-PMPs with excellent softness demonstrated superior tumor accumulation, deep tumor penetration and enhanced internalization into tumor cells to efficiently kill tumor cells. Through interacting with TLR-4 receptors on tumor cells to reduce platelet activation, DOX@3D-PMPs significantly inhibited platelet-promoted tumor proliferation and metastasis to enhance chemotherapeutic efficacy.

Herein, we developed novel hybrid MPs by fusing 3D-MPs with inactivated platelet membranes (3D-PMPs) to load anticancer drug doxorubicin (DOX@3D-PMP, Fig. 1A). Leveraging the unique softness of 3D-MPs, DOX@3D-PMPs demonstrated superior tumor accumulation, deep tumor penetration, and enhanced internalization into tumor cells to efficiently kill tumor cells. Importantly, 3D-PMPs, enriched with platelet-related ligands, functioned as platelet decoys by interacting with receptors on tumor cells to reduce platelet activation, thereby inhibiting platelet-promoted tumor proliferation and metastasis (Fig. 1B). This approach offers a promising strategy for enhancing the therapeutic efficacy of cancer treatments.

## 2 Materials and methods

### 2.1 Materials

RPMI 1640, Dulbecco's modified Eagle's medium (DMEM), fetal bovine serum (FBS), collagenase type I, l-glutamine, B27 supplement (vitamin A-free), penicillin and streptomycin were sourced from Gibco BRL/Life Technologies (Grand Island, NY, USA). Mouse epithelial growth factor was obtained from PeproTech (Cranbury, NJ, USA). Dispase II proteinase and prostaglandin E1 were purchased from Sigma–Aldrich (St. Louis, MO, USA). Doxorubicin hydrochloride was supplied by Beijing HuaFeng United Technology Co., Ltd. (Beijing, China), while Doxil was sourced from Fudan-Zhangjiang Bio-Pharmaceutical (Shanghai, China). Fibrinogen and thrombin were acquired from Searun Holdings Company (Freeport, TX, USA). All other reagents used in this study were of analytical grade and purchased from Sinopharm Chemical Reagent Co., Ltd. (Shanghai, China). Antibodies used in this study were purchased from BioLegend (San Diego, CA, USA).

### 2.2 Cell culture and animals

The 4T1 cells and B16-F10 cells were acquired from the Type Culture Collection of the Chinese Academy of Sciences (Shanghai, China), while the luciferase-expressing 4T1 (4T1-Luc) cell line was generously provided by Dr. Yinsong Wang from Tianjin Medical University (Tianjin, China). 4T1, 4T1-Luc, and B16-F10 cells were cultured in RPMI-1640 supplemented with 10% FBS and 1% penicillin–streptomycin at 37 °C in 5% CO_2_. TRCs were isolated using soft 3D fibrin gels (around 90 Pa) as previously described^28^. Briefly, 4T1 or B16-F10 cells were embedded in fibrin gels, cultured for 5 days, and spheroids were enzymatically dissociated to obtain single-cell TRCs for subsequent experiments. For platelet isolation, whole blood was collected from BALB/c mice and subjected to centrifugation at 180 × *g* (Legend Micro 21R, Thermo Fisher Scientific, Waltham, MA, USA) for 20 min to obtain platelet-rich plasma, and then centrifuged at 1800×*g* (Thermo Fisher Scientific) for 10 min to isolate the platelets.

Female BALB/c and male C57BL/6 mice, weighing between 18 and 20 g, were obtained from Beijing Vital River Laboratory Animal Technology Co., Ltd. (Beijing, China). The mice were maintained under standard SPF conditions. To establish the orthotopic 4T1 tumor-bearing mouse model, 5 × 10^5^ 4T1 cells were injected into the right fourth mammary fat pad of the female BALB/c mice. For the post-surgical tumor model, approximately 90% of the tumor was surgically removed when it reached about 200 mm^3^. To establish the experimental metastatic B16-F10 tumor-bearing mouse model, 5 × 10^5^ B16-F10 cells were injected into the male C57BL/6 mice through the tail vein. All animal experiments were performed following the ethical guidelines and approved protocols of the Institutional Animal Care and Use Committee at Tongji Medical College, Huazhong University of Science and Technology (Wuhan, China), the approval number is HUST-IACUC-2025-0018.

### 2.3 Preparation and characterization of DOX@3D-PMPs

4T1 TRCs or B16-F10 TRCs were subjected to ultraviolet irradiation (300 J/m) for 1 h, and following a 12-h incubation, the supernatants were collected and centrifuged at 600×*g* (Thermo Fisher Scientific) for 10 min, followed by a second centrifugation at 14,000×*g* (Thermo Fisher Scientific) for 2 min to remove any cell debris. The resulting supernatants were then subjected to a final centrifugation at 14,000×*g* (Thermo Fisher Scientific) for 1 h to isolate 3D-MPs. The pellet was washed three times with PBS and resuspended in PBS for subsequent experiments.

3D-MPs were generated from 4T1 or B16-F10 TRCs by ultraviolet irradiation (300 J/m, 1 h) followed by a 12 h incubation. The supernatants were sequentially centrifuged (600 × *g*, 10 min; 14,000 × *g*, 2 min; 14,000 × *g*, 1 h) and the pellets were washed and resuspended in PBS. Platelets were washed with PBS containing 1 mmol/L EDTA and 2 μmol/L prostaglandin E1, resuspended in PBS with 1 mmol/L EDTA and protease inhibitors, and subjected to five liquid-nitrogen and 37 °C freeze-thaw cycles. Platelet membranes were collected by centrifugation (4000 × *g*, Thermo Fisher Scientific, 5 min). To prepare 3D-PMPs, 3D-MPs and platelet membranes were mixed at a 3:1 protein ratio, sonicated for 5 min, incubated at 37 °C for 30 min, and centrifuged at 20,000 × *g* (Thermo Fisher Scientific) for 60 min to obtain hybrid vesicles. The fragmented platelet membranes (PNPs) were prepared using the same membrane-coating procedure.

For DOX loading, 100 μg of 3D-PMPs, PNPs, or 3D-MPs were incubated with 100 μg DOX in 2 mL PBS at 37 °C for 4 h, followed by centrifugation (20,000 × *g*, Thermo Fisher Scientific, 1 h) and washing to obtain DOX-loaded PNPs (DOX@PNPs), 3D-MPs (DOX@3D-MPs) and 3D-PMPs (DOX@3D-PMPs). DOX loading content was quantified by high-performance liquid chromatography (HPLC, Agilent Technologies, Santa Clara, CA, USA). Hydrodynamic diameter and zeta potential were measured using a Zetasizer Nano ZS90 (Malvern Instruments, Malvern, UK). Morphology was examined by Transmission electron microscope (TEM, Tecnai G2, FEI Company, Hillsboro, OR, USA) at voltages of 20 or 80 kV. Particle Young's modulus was measured in PBS at room temperature using atomic force microscopy (AFM, Multimode 8, Bruker, Billerica, MA, USA). Membrane fusion between 3D-MPs and platelet membranes was confirmed by DiO/DiD dual-labeling and confocal imaging (FV3000, Olympus, Tokyo, Japan).

### 2.4 Cellular uptake and viability assay

3 × 10^5^ 4T1 cells were seeded into 6-well plates and incubated overnight. The cells were then exposed to free DOX, DOX@3D-MPs, DOX@PNPs, or DOX@PNPs at different DOX concentrations for 4 h, or with 1 μg/mL DOX equivalent for different incubation times. After treatment, cells were washed three times with PBS, and intracellular DOX fluorescence was quantified by flow cytometry (CytoFLEX S, Beckman Coulter, Brea, CA, USA). For viability evaluation, 8 × 10^4^ 4T1 cells were seeded in 96-well plates and incubated overnight, followed by exposure to free DOX, DOX@3D-MPs, DOX@PNPs, or DOX@PNPs at different DOX concentrations for 24 h. Cell viability was assessed using a Cell-Counting Kit-8 assay (Donjindo, Kyushu, Japan). The absorbance at 450 nm was recorded using a FlexStation 3 microplate reader (Molecular Devices, San Jose, CA, USA).

### 2.5 Tumor penetration in 3D tumor spheroids

3D tumor spheroids of 4T1 cells were generated following established protocols^27^. Once the spheroids reached about 120‒150 μm in diameter, they were treated with free DOX, DOX@3D-MPs, DOX@PNPs or DOX@3D-PMPs at a DOX concentration of 1 μg/mL for 4 h. After treatment, the spheroids were washed with PBS, fixed with 4% paraformaldehyde, and mounted on confocal dishes. DOX penetration was evaluated using Z-stack imaging (5 μm step size) acquired on an FV3000 confocal microscope.

### 2.6 *In vivo* biodistribution and tumor penetration

For DOX biodistribution, 4T1 tumor-bearing mice were intravenously administrated with free DOX, DOX@3D-MPs, DOX@PNPs, or DOX@3D-PMPs at a DOX dosage of 0.8 mg/kg, or Doxil at a higher DOX dosage of 4 mg/kg. At 24 h post-treatment, mice were euthanized and major organs (heart, liver, spleen, lung and kidney) and tumors were harvested. The tissues were homogenized in 100 μL of PBS, followed by the addition of 200 μL of methanol (4 °C, 30 min, dark), followed by centrifugation (10,000 × *g*, Thermo Fisher Scientific, 10 min). DOX fluorescence in the supernatant (100 μL) was quantified using a FlexStation 3 plate reader (Molecular Devices). Calibration curves were obtained by spiking known DOX concentrations into matched tissues and processing them in parallel. For *in vivo* tumor penetration, tumor tissues collected 24 h after administration (same dosing regimen as above) were washed, cryosectioned, and stained with FITC-anti-CD31 antibody (BioLegend, cat. No 102405, clone 390, 1/50 dilution) at 37 °C for 30 min. After PBS washing, vascular structures and DOX distribution were imaged on an FV3000 confocal microscope (Olympus).

### 2.7 Interaction of platelets with tumor cells

1 × 10^5^ 4T1 cells were treated with PBS, 3D-MPs, PNPs, 3D-PMPs or an anti-TLR-4 antibody (*α*TLR-4)-pretreated 3D-PMPs (3D-PMPs+*α*TLR-4) at the protein concentration of 10 μg/mL for 4 h. These treated cells were then co-incubated with DiD-labeled platelets at a 4T1 cell-to-platelets number ratio of 1:1000 for 4 h. These cells were fixed with 4% paraformaldehyde, washed with PBS, and the proportion of DiD^+^ 4T1 cells was analyzed by CytoFLEX S flow cytometry (Beckman Coulter). For confocal microscopic analysis, these fixed cells were washed with PBS and visualized using an FV3000 confocal microscope (Olympus).

### 2.8 *In vitro* inhibition of tumor invasion

For wound-healing assays, 1.5 × 10^5^ 4T1 cells were seeded into scratch test molds and allowed to adhere overnight. Cells were treated with 3D-MPs, PNPs, 3D-PMPs, or *α*TLR4-pretreated 3D-PMPs (10 μg/mL protein, 4 h), washed with PBS, and then cultured in serum-free medium with or without platelets (4T1-to-platelet number ratio: 1:1000) for 18 h. Scratch closure was imaged using an inverted microscope and quantified with ImageJ software.

For transwell invasion assays, 6 × 10^4^ 4T1 cells were seeded into the upper chamber of transwell inserts in serum-free medium and incubated overnight. The same treatments were applied (PBS, 3D-MPs, PNPs, 3D-PMPs, or 3D-PMPs+*α*TLR4; 10 μg/mL protein, 4 h), followed by PBS washing and incubation with or without platelets at a 4T1 cell-to-platelets number ratio of 1:1000. Medium containing 10% FBS was added to the lower chamber as a chemoattractant. After 18 h, invaded cells were fixed with 4% paraformaldehyde, stained with 0.1% crystal violet, and quantified microscopically.

### 2.9 *In vivo* inhibition of tumor metastasis

Transgenic zebrafish (Tg[kdrl:mcherry]) were kindly provided by Prof. Mugen Liu (Huazhong University of Science and Technology, Wuhan, China). For the zebrafish metastasis model, DIO-labeled 4T1 cells were pretreated with PBS, 3D-MPs, PNPs, or 3D-PMPs (10 μg/mL protein, 4 h). Treated cells (200 cells per embryo), with or without platelets (4T1-to-platelet ratio: 1:1000), were injected into the yolk sac of zebrafish embryos at 48 h post-fertilization. Embryos were maintained at 34 °C for 4 days, after which migration of 4T1 cells to the tail region was imaged by FV3000 confocal microscopy and quantified using ImageJ software.

For the mouse metastasis model, 1 × 10^5^ 4T1-Luc cells pretreated with PBS, 3D-MPs, PNPs, 3D-PMPs, or 3D-PMPs+*α*TLR4 (10 μg/mL protein, 4 h) were co-injected with or without platelets at a 4T1 cell-to-platelets number ratio of 1:1000 *via* tail vein into BALB/c mice. Tumor progression was monitored by bioluminescence imaging before injection and again at Day 5. For lung metastasis evaluation, lungs were collected on Day 15, imaged using a bioluminescence system, fixed in Bouin's solution for 12 h at room temperature, and metastatic nodules were counted. Lungs were subsequently processed for Hematoxylin–eosin (H&E) staining.

### 2.10 *In vivo* antitumor effects and platelet analysis

Therapeutic performance and safety were evaluated in orthotopic and post-surgical 4T1-Luc tumor-bearing mice. Mice received intravenous injections of PBS, 3D-MPs, PNPs, 3D-PMPs, free DOX, DOX@3D-MPs, DOX@PNPs or DOX@3D-PMPs (0.5 mg DOX/kg), while Doxil was administered at 4 mg DOX/kg. Treatments were given every 3 days for a total of 4‒5 doses. Tumor volume and body weight were monitored daily. At the study endpoint, tumors and major organs (heart, liver, spleen, lung and kidney) were collected for H&E staining, and the remaining mice were followed for survival. For bioluminescence monitoring, D-luciferin (150 mg/kg) was injected intraperitoneally and imaged 5 min later using an IVIS Lumina II system (Caliper Life Sciences, Hopkinton, MA, USA) every 5 days to track tumor progression. For lung metastasis evaluation, lungs were harvested, fixed in Bouin's solution for 12 h at room temperature, and metastatic nodules were counted before H&E staining.

Tumors were excised, minced, and digested in RPMI-1640 containing DNase I (5 μg/mL) and collagenase I (0.8 mg/mL) at 37 °C for 1 h. Tumor suspensions were centrifuged (350×*g*, Thermo Fisher Scientific, 5 min), washed, treated with RBC lysis buffer, and filtered twice through a 40 μm mesh to obtain single-cell suspensions. Cells were stained for 30 min with fluorophore-conjugated anti-CD41 antibody (BioLegend, cat. No. 133904, clone MWReg30, 1/200 dilution), anti-CD62P antibody (BioLegend, cat. No. 148310, clone RMP-1, 1/100 dilution), and anti-CD63 antibody (BioLegend, cat. No. 1439906, clone NCG-2, 1/50 dilution), following manufacturer instructions, and analyzed by flow cytometry.

### 2.11 Statistical analysis

All experiments were independently repeated at least three times. Statistical analysis was performed using GraphPad Prism 8.4 software (GraphPad software, USA). One-way ANOVA or two-way ANOVA was used for multiple comparisons, unless otherwise specified. Data are expressed as mean ± standard deviation (SD), and statistical significance was defined as *P* < 0.05.

## 3 Results

### 3.1 Preparation and characterization of DOX@3D-PMPs

**Figure 2:**
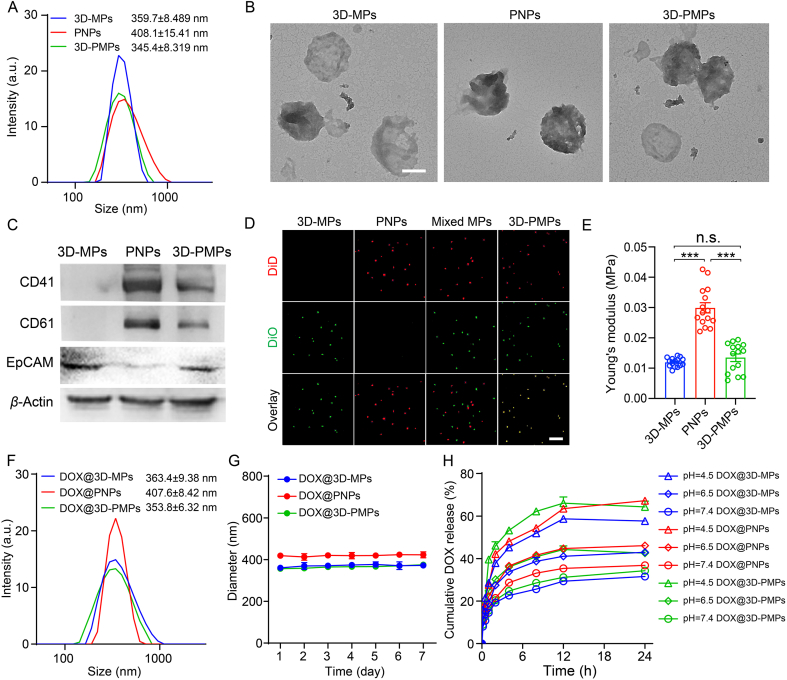
Characterization of DOX@3D-PMPs. (A) Hydrodynamic diameter of 3D-MPs, PNPs and 3D-PMPs measured by DLS. (B) Morphology of 3D-MPs, PNPs and 3D-PMPs measured by TEM. Scale bar = 200 nm. (C) Western blotting analysis of CD41, CD61 and EpCAM on 3D-MPs, PNPs and 3D-PMPs. (D) Co-localization of DiD-labeled 3D-MPs (green) and DiO-labeled platelet membranes (red) within 3D-PMPs by confocal microscopy. Scale bar = 60 μm. (E) Young's modulus of 3D-MPs, PNPs and 3D-PMPs measured by AFM. (mean ± SD, *n* = 15), ∗∗∗*P* < 0.001 *vs*. indicated; n.s., non-significant (one-way ANOVA with Tukey's HSD *post hoc* test). (F) Hydrodynamic diameter of DOX@3D-MPs, DOX@PNPs and DOX@3D-MPs by DLS. (G) Hydrodynamic diameter of DOX@3D-PMPs after incubation in PBS at various time points by DLS. (mean ± SD, *n* = 3). (H) *In vitro* DOX release profiles of DOX@3D-PMPs, DOX@PNPs and DOX@3D-MPs in PBS at different pH levels using a dialysis bag method. (mean ± SD, *n* = 3).

To acquire TRC- and platelet-derived hybrid MPs, 3D-MPs were fused with PNPs using sonication. Our previous work demonstrated that the softness of 3D-MPs significantly influenced their drug delivery efficiency. To achieve a balance between softness and platelet decoy efficiency, the ratio of 3D-MPs to fragmented platelet membranes was optimized to 3:1, as determined by measuring the Young's modulus of 3D-PMPs using AFM (Supporting Information Fig. S1). Dynamic light scattering (DLS) analysis showed that the diameter of 3D-PMPs, 3D-MPs and PNPs was approximately 345 nm, 359 nm, and 408 nm, respectively (Fig. 2A), with a comparable zeta potential of about –10.8 mV. TEM revealed their similar monodisperse and intact vesicular structures (Fig. 2B). Western blotting analysis confirmed the presence of CD41 and CD61, specific platelet biomarkers, as well as EpCAM, which is abnormally expressed in 4T1 cells, in 3D-PMPs (Fig. 2C), indicating successful fusion of 3D-MPs and the fragmented platelet membranes. The clear overlay of DiD-labeled 3D-MPs and DiO-labeled platelet membranes further validated the successful formation of 3D-PMPs through the sonication process, whereas a simple mixture of 3D-MPs and platelet membranes did not achieve this (Fig. 2D). AFM analysis indicated that the Young's modulus of 3D-PMPs was similar to that of 3D-MPs but significantly lower than that of PNPs (Fig. 2E), suggesting the potential tumor-targeting ability of 3D-PMPs.

Furthermore, PNPs, 3D-MPs and 3D-PMPs were incubated with DOX to create DOX@PNPs, DOX@3D-MPs and DOX@3D-PMPs, respectively. The drug loading efficiency for PNPs, 3D-MPs and 3D-PMPs was measured at 7 μg DOX per 100 μg protein (MP quantification based on total protein content) using HPLC, indicating that the fusion with PNPs did not significantly alter the drug loading capacity of 3D-MPs. Moreover, the addition of DOX did not remarkably affect the diameter of PNPs, 3D-MPs, or 3D-PMPs (Fig. 2F). DOX@3D-PMPs maintained a consistent size after 7 days of incubation in PBS (Fig. 2G), indicating their good stability. *In vitro* drug release analysis demonstrated that DOX@PNPs, DOX@3D-MPs and DOX@3D-PMPs exhibited pH-dependent DOX release, with the release rate reaching approximately 67% at pH 4.5 over 24 h (Fig. 2H), suggesting that DOX@3D-PMPs might efficiently release DOX within lysosomes after being internalized by tumor cells.

### 3.2 Enhanced tumor accumulation, deep penetration and efficient tumor cellular uptake of DOX@3D-PMPs

**Figure 3:**
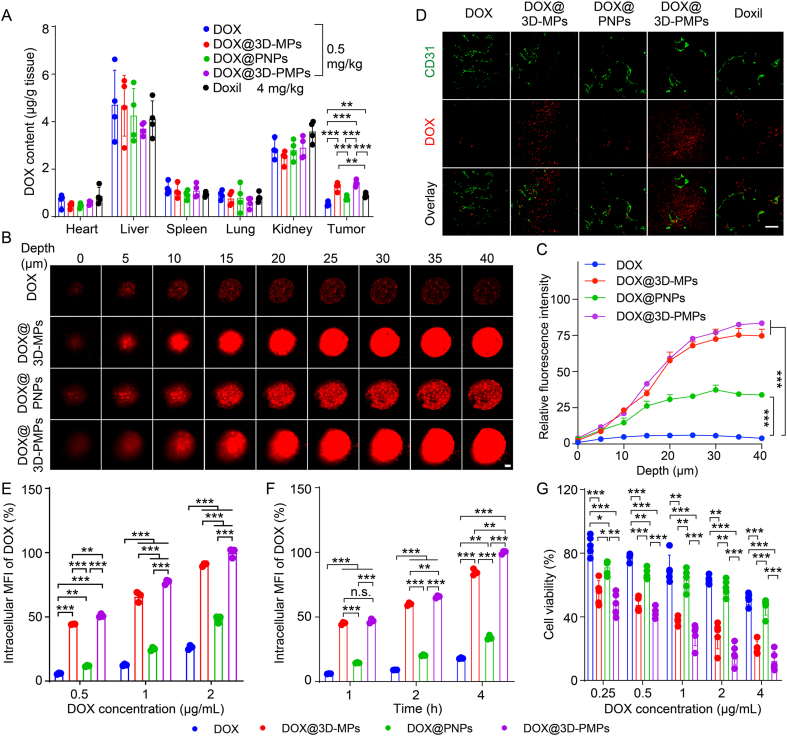
DOX@3D-PMPs enhance tumor accumulation, deep penetration and cellular uptake. (A) DOX content in tumors and major organs of orthotopic 4T1 tumor-bearing mice 24 h after intravenous administration of free DOX, DOX@3D-MPs, DOX@PNPs, DOX@3D-PMPs, or Doxil. (mean ± SD, *n* = 4). (B) DOX distribution in tumor spheroids after indicated treatments for 24 h. Scale bar = 50 μm. (C) Quantitative analysis of DOX fluorescence in tumor spheroids after treatments described in (B). (mean ± SD, *n* = 3). (D) DOX distribution in tumor tissues of orthotopic 4T1 tumor-bearing mice 24 h post-injection. Tumor vessels were labeled with FITC-anti-CD31. Scale bar = 5 μm. (E, F) Relative mean fluorescence intensity (MFI) of DOX in 4T1 cells after indicated treatments for 4 h (E) or at different time intervals (F) (mean ± SD, *n* = 3). (G) The viability of 4T1 cells after treatments indicated at varying DOX concentrations for 24 h (mean ± SD, *n* = 5). ∗*P* < 0.05, ∗∗*P* < 0.01, ∗∗∗*P* < 0.001 *vs*. indicated (two-way ANOVA followed by Tukey's multiple comparisons *post hoc* test for A and E–G; one-way ANOVA followed by Tukey's HSD *post hoc* test for C).

An ideal system for delivering chemotherapeutic agents should exhibit enhanced tumor accumulation, deep penetration into tumor tissues, and efficient uptake by tumor cells^25^^,^^27^. Our previous work demonstrated that the unique softness of 3D-MPs significantly improved drug delivery efficiency^28^. Given that 3D-PMPs possessed similar Young's modulus, the tumor accumulation of DOX@3D-PMPs was first evaluated in mice bearing orthotopic 4T1 breast tumors. These mice were administrated intravenously with free DOX, DOX@3D-MPs, DOX@PNPs or DOX@3D-PMPs at a DOX dosage of 0.5 mg/kg, or Doxil at a higher dosage of 4 mg/kg. 24 h post-injection, the DOX levels in the tumors and major organs were evaluated (Fig. 3A). Similar to DOX@3D-MPs, DOX@3D-PMPs accumulated more in tumor tissues, approximately 2.6-, 1.8- and 1.6-fold higher than free DOX, DOX@PNPs and high-dose Doxil, respectively, indicating that DOX@3D-PMPs have an enhanced ability to accumulate in tumors. Furthermore, long-term distribution results confirmed sustained tumor retention and rapid clearance in hepatic and renal tissues. Consistently, DiR-labeled 3D-PMPs exhibited durable tumor accumulation over 24‒48 h while showing time-dependent reduction of hepatic and renal fluorescence, indicating minimal long-term retention in normal organs (Supporting Information Fig. S2).

The tumor penetration of DOX@3D-PMPs was further evaluated. Tumor spheroids were exposed to free DOX, DOX@3D-MPs, DOX@PNPs or DOX@3D-PMPs, and the DOX fluorescence intensity at various depths was observed. Consistently, significantly higher DOX fluorescence intensity was detected in DOX@3D-PMPs- and DOX@3D-MPs-treated groups, even at a depth of 40 μm (Fig. 3B and C), indicating the deep tumor penetration capacity of DOX@3D-PMPs. This deep tumor penetration was further confirmed in orthotopic 4T1 tumor-bearing mice. In contrast to the strong co-localization of DOX and anti-CD31-labeled tumor vessels in mice intravenously injected with free DOX, more DOX was extravasated from tumor vessels in the DOX@3D-PMPs-treated group, approximately 17.3-, 13.9-, and 3.4-fold higher than in free DOX-, DOX@PNPs- and high-dose Doxil-treated groups, respectively (Fig. 3D; Supporting Information Fig. S3).

After achieving efficient tumor accumulation and deep penetration, DOX@3D-PMPs need to be effectively internalized by tumor cells to exert their antitumor effects. As anticipated, free DOX, DOX@3D-MPs, DOX@PNPs, and DOX@3D-PMPs were internalized into 4T1 cells in a dose- and time-dependent manner (Fig. 3E and F). Notably, DOX@3D-MPs, DOX@PNPs and DOX@3D-PMPs exhibited enhanced cellular uptake compared to free DOX, with DOX@3D-PMPs achieving the highest level of internalization among all groups (Fig. 3E and F). Consistently, DOX@3D-PMPs showed strong cytotoxicity against 4T1 cells (Fig. 3G). To further elucidate their intracellular fate, we observed that DOX@3D-PMPs traffic into lysosomes, where the vesicular structures remain confined, while DOX gradually escapes into the cytosol and ultimately accumulates in the nucleus (Supporting Information Fig. S4). This release behavior is consistent with earlier findings showing that TRC-derived MPs can subtly modulate lysosomal acidification and membrane permeability, thereby reducing drug entrapment and facilitating lysosomal escape^30^.

### 3.3 Efficient suppression of platelet-mediated tumor cell growth and migration by 3D-PMPs

**Figure 4:**
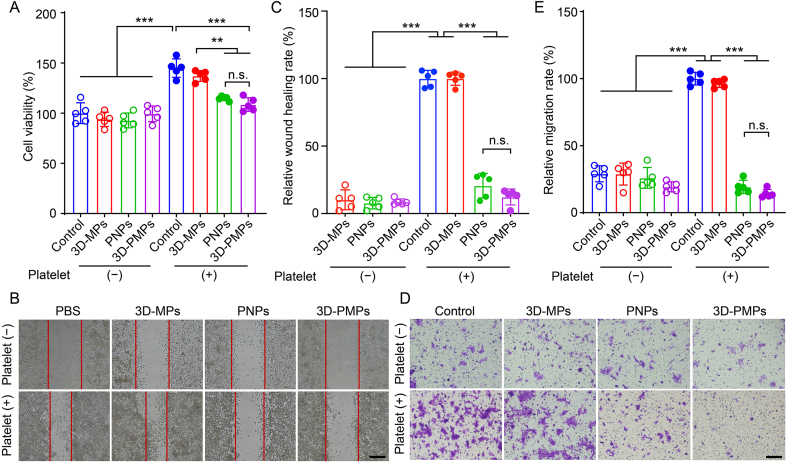
3D-PMPs inhibit platelet-promoted tumor cell growth and migration *in vitro*. (A) Viability of 4T1 cells pretreated with PBS, 3D-MPs, PNPs or 3D-PMPs at a protein concentration of 10 μg/mL for 4 h, followed by co-incubation with or without platelets for 18 h (mean ± SD, *n* = 5). (B) Representative wound-healing images of 4T1 cells after treatments indicated in (A). Scale bar = 200 μm. (C) Quantification of wound closure rate of 4T1 cells after treatments described in (A). (mean ± SD, *n* = 5). (D) Representative images of the migratory 4T1 cells after treatments described in (A) by transwell assay. Scale bar = 200 μm. (E) Quantification of the migratory 4T1 cells after treatments described in (A) by transwell assay. (mean ± SD, *n* = 5). ∗∗*P* < 0.01, ∗∗∗*P* < 0.001 *vs*. indicated; n.s., non-significant (one-way ANOVA followed by Tukey's HSD *post hoc* test for A, C and E).

Platelets play a critical role in promoting tumor cell proliferation, invasion, and metastasis^18^^,^^19^. Since 3D-PMPs were enriched with platelet-associated proteins, we investigated whether 3D-PMPs could act as platelet decoys to inhibit platelet-induced tumor cell behaviors. After 4T1 cells were pretreated with PBS, 3D-MPs, PNPs, or 3D-PMPs and then co-incubated with or without platelets, cell viability of 4T1 cells was assessed (Fig. 4A). Expectedly, 3D-MPs, PNPs, and 3D-PMPs alone did not obviously affect the proliferation of 4T1 cells, while the platelets significantly promoted their proliferation. Although 3D-MPs had minimal impact on platelet-driven 4T1 cell proliferation, both PNPs and 3D-PMPs efficiently inhibited this effect, suggesting that 3D-PMPs, functioning as platelet decoys, effectively disrupted platelet-mediated tumor cell proliferation. This enhanced suppression is further supported by our additional experiments demonstrating a clear synergistic effect between DOX-mediated cytotoxicity and the ability of 3D-PMPs to block platelet-derived pro-tumorigenic signaling (Supporting Information Fig. S5). Furthermore, the effects of 3D-PMPs on platelet-induced migration of 4T1 cells were evaluated using a scratch wound healing assay. Consistently, platelets significantly promoted the migration of 4T1 cells. However, PNPs and 3D-PMPs, but not 3D-MPs, effectively inhibited platelet-driven migration (Fig. 4B and C). Notably, the wound healing rate was significantly reduced to about 12% in the 3D-PMPs-treated group compared with the PBS-treated group, demonstrating the strong inhibitory effects of 3D-PMPs on platelet-induced tumor cell migration. The inhibition of platelet-induced tumor cell migration by 3D-PMPs was further validated using a transwell migration assay (Fig. 4D and E).

**Figure 5:**
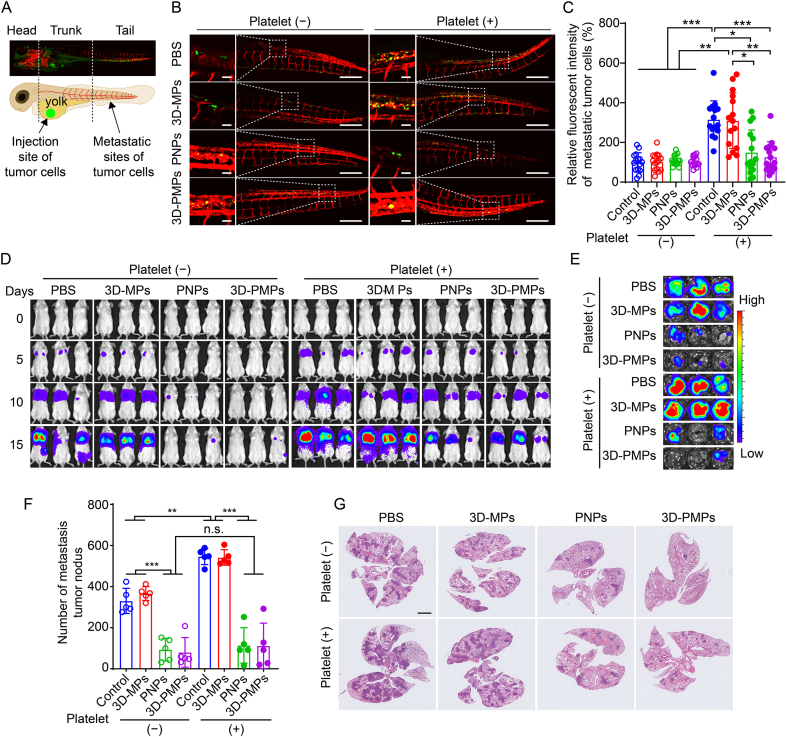
3D-PMPs inhibit platelet-induced tumor metastasis *in vivo*. (A) Schematic diagram of tumor metastasis model using Tg(kdrl:mcherry) transgenic zebrafish. (B) Representative fluorescence images of CFSE-labeled 4T1 cells in the tail region of zebrafish at 4 days after co-injection of pretreated 4T1 cells with PBS, 3D-MPs, PNP or 3D-PMPs at a protein concentration of 10 μg/mL for 4 h with or without platelets into the zebrafish yolk sacs. Scale bar = 30 μm (left) or 200 μm (right). (C) Quantification of CFSE-labeled 4T1 cells in the tail region of zebrafish after treatments described in (B). (mean ± SD, *n* = 15). (D) *In vivo* bioluminescence images of BALB/c mice injected intravenously with 4T1-Luc cells pretreated as above, with or without platelets. (E) *Ex vivo* bioluminescence images of lungs collected on Day 15 (D). (F) Quantification of lung metastatic nodules on Day 15 (D). (mean ± SD, *n* = 5). (G) Representative H&E-stained lung sections on Day 15 (D). (Scale bar = 2 mm). ∗*P* < 0.05, ∗∗*P* < 0.01, ∗∗∗*P* < 0.001 *vs*. indicated; n.s., non-significant (one-way ANOVA followed by Tukey's HSD *post hoc* test for C and F).

To explore the inhibitory effects of 3D-PMPs on platelet-induced tumor metastasis *in vivo*, we developed a novel tumor metastasis model using Tg(kdrl:mcherry) transgenic zebrafish, which allows precise visualization of metastatic progression. CSFE-labeled 4T1 cells pretreated with PBS, 3D-MPs, PNPs or 3D-PMPs were injected into the yolk sacs of the zebrafish either in the presence or absence of platelets, and the metastatic behaviors were evaluated by monitoring CFSE fluorescence in the tail region (Fig. 5A). As expected, co-injection of platelets significantly enhanced the colonization of 4T1 cells in the zebrafish tail. While 3D-MPs, PNPs, or 3D-PMPs alone did not significantly affect the metastasis of 4T1 cells, the PNPs- and 3D-PMPs-treated groups showed a marked reduction in metastatic 4T1 cells in the zebrafish tails compared to the PBS-treated group when platelets were co-injected (Fig. 5B and C), demonstrating that 3D-PMPs might act as platelet decoys to inhibit platelet-promoted tumor metastasis. The anti-metastatic potential of 3D-PMPs was further investigated in a mouse model bearing 4T1-Luc tumors. 4T1-Luc cells were pretreated with PBS, 3D-MPs, PNPs or 3D-PMPs and then co-injected intravenously with or without platelets into BALB/c mice. *In vivo* bioluminescence imaging revealed a time-dependent increase in signal intensity in PBS-treated group (Fig. 5D), indicating significant metastasis of 4T1-Luc tumors. Co-injection of platelets markedly accelerated the metastasis of 4T1-Luc tumors (Fig. 5D). In contrast, PNPs and 3D-PMPs, but not 3D-MPs, effectively suppressed tumor metastasis, regardless of platelet presence (Fig. 5D). *Ex vivo* imaging of lung tissues confirmed these findings, showing minimal signal intensity in the PNP- and 3D-PMPs-treated groups (Fig. 5E). Additionally, the number of metastatic nodules in the lungs was significantly reduced in the 3D-PMP-treated group, as evidenced by both nodule counting (Fig. 5F) and H&E staining (Fig. 5G). These results highlighted the strong anti-metastatic effects of 3D-PMPs, particularly in countering platelet-enhanced tumor spread.

### 3.4 TLR-4 involvement in the 3D-PMPs-mediated disruption of platelet-tumor cell interaction

**Figure 6:**
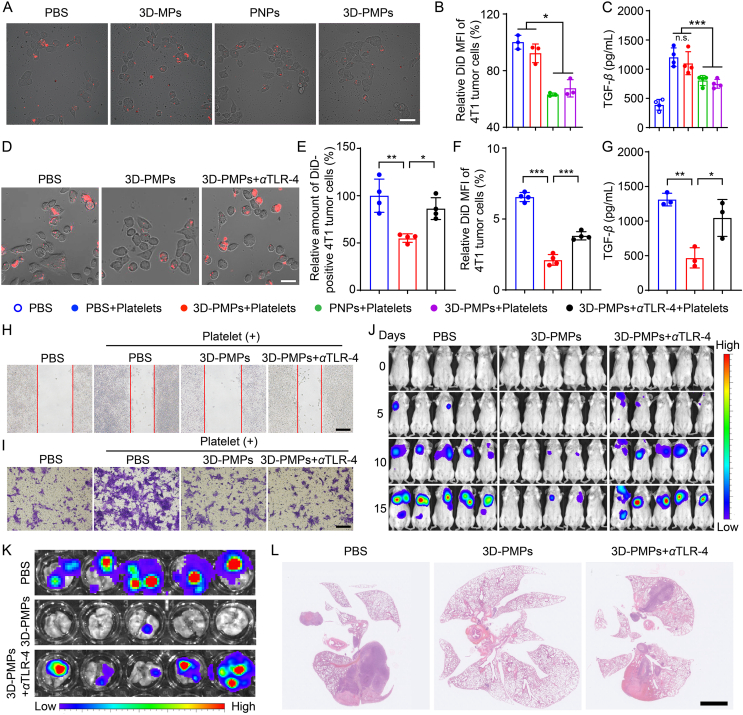
TLR-4 mediates the inhibitory effect of 3D-PMPs on platelet-induced tumor metastasis. (A) Confocal images of 4T1 cells pretreated with PBS, 3D-MPs, PNPs or 3D-PMPs at a protein concentration of 10 μg/mL for 4 h, followed by incubation with DiD-labeled platelets for 4 h. Scale bar = 20 μm. (B) Flow cytometric analysis of DiD MFI of 4T1 cells after treatments described in (A). (mean ± SD, *n* = 3). (C) TGF-*β* levels in the supernatants of 4T1 cells treated as in (A) and subsequently incubated with platelets for 4 h (mean ± SD, *n* = 4). (D) Confocal images of 4T1 cells pretreated with PBS, 3D-PMPs or *α*TLR-4-blocked 3D-PMPs for 4 h, followed by incubation with DiD-labeled platelets for 4 h. Scale bar = 20 μm. (E, F) Flow cytometric analysis of DiD-positive 4T1 cells (E) and DiD MFI (F) after treatments described in (D). (mean ± SD, *n* = 4). (G) TGF-*β* levels in the supernatants of 4T1 cells treated as in (D) and incubated with platelets for 4 h (mean ± SD, *n* = 3). (H, I) Representative wound healing (H) and transwell migration (I) images of 4T1 cells pretreated with PBS, 3D-PMPs or *α*TLR-4-blocked 3D-PMPs, followed by incubation with or without platelets for 18 h. Scale bar = 200 μm. (J) *In vivo* bioluminescence images of BALB/c mice injected intravenously with 4T1-Luc cells pretreated as in (H, I) with or without platelets. (K, L) *Ex vivo* bioluminescence (K) and H&E-stained lung sections (L) at 15 days after intravenous injection as indicated in (J). Scale bar = 2 mm. ∗*P* < 0.05, ∗∗*P* < 0.01, ∗∗∗*P* < 0.001 *vs*. indicated (one-way ANOVA followed by Tukey's HSD *post hoc* test for B, C and E–G).

Previous studies have demonstrated that platelets can adhere to tumor cells through specific surface proteins, such as CD97^31^ and CCR5^32^, triggering intracellular signaling pathways that promote tumor proliferation, metastasis and recurrence. Given the presence of these key proteins on 3D-PMPs, we investigated their effects on platelet-tumor cell interaction. 4T1 cells were pretreated with PBS, 3D-MPs, PNPs or 3D-PMPs for 4 h, followed by co-incubation with DiD-labeled platelets for an additional 4 h. Confocal microscopic images revealed that 4T1 cells pretreated with PBS or 3D-MPs displayed the highest levels of red fluorescence from platelets (Fig. 6A), indicating strong platelet adhesion. In contrast, the PNPs- or 3D-PMPs-treated group showed significantly reduced red fluorescence. Flow cytometric analysis confirmed a significant decrease in platelet adhesion to PNPs- and 3D-PMPs-treated 4T1 cells compared to PBS- or 3D-MPs-treated group (Fig. 6B), corresponding to a remarkable decrease in TGF-*β* release (Fig. 6C), a critical factor in promoting tumor metastasis. These results suggest that 3D-PMPs might act as platelet decoys to inhibit platelet-tumor cell interaction.

To further determine whether the mechanical softness of 3D-PMPs contributes to their ability to block platelet-tumor interactions, we generated a mechanically “non-soft” control by treating 3D-PMPs with jasplakinolide (Jasp), an actin-stabilizing agent known to increase cytoskeletal stiffness^28^. Compared with their soft counterparts, Jasp-treated 3D-PMPs displayed markedly reduced internalization by 4T1 cells and showed an increased platelet adhesion on 4T1 cells (Supporting Information Fig. S6), indicating that particle softness is essential for efficient tumor adhesion and effective masking of platelet-tumor interactions.

TLR-4, expressed on the platelet membrane, is known to mediate platelet-tumor cell interaction, influencing their adhesion and aggregation^33^^,^^34^. To explore the role of TLR-4 in the ability of 3D-PMPs to inhibit this interaction, TLR-4 expression on 3D-PMPs and PNPs was first confirmed by western blotting analysis (Supporting Information Fig. S7). Subsequently, 3D-PMPs were pretreated with *α*TLR-4 and then incubated with 4T1 cells in the presence of DiD-labeled platelets. Expectedly, *α*TLR-4 significantly impaired the function of 3D-PMPs, leading to increased platelet adhesion to 4T1 cells, as evidenced by strong red fluorescence in 4T1 cells incubated with *α*TLR-4-pretreated 3D-PMPs (3D-PMPs+*α*TLR-4) (Fig. 6D), higher numbers of adherent platelets (Fig. 6E and F), and elevated TGF-*β* secretion (Fig. 6G). These findings confirmed that the inhibition of platelet-tumor cell interaction by 3D-PMPs was largely dependent on the presence of TLR-4. We also examined whether other platelet-tumor adhesion receptors, such as P-selectin and glycoprotein IIb/IIIa (GPIIb/IIIa), contribute to the function of 3D-PMPs. Blocking P-selectin with KF38789 inhibitor or GPIIb/IIIa with Tirofiban inhibitor did not affect 3D-PMP uptake by 4T1 cells, whereas TLR-4 blockade markedly reduced it (Supporting Information Fig. S8). This is likely because P-selectin and GPIIb/IIIa operate mainly in activated platelets, while the platelet membranes used here are non-activated with low P-selectin exposure and inactive GPIIb/IIIa, leaving TLR-4 as the predominant mediator of 3D-PMP-tumor cell interaction.

Furthermore, the involvement of TLR-4 in 3D-PMPs-mediated inhibition of platelet-promoted tumor metastasis was evaluated. Consistently, blocking TLR-4 significantly abolished the 3D-PMPs-induced inhibition of 4T1 cell migration and invasion (Fig. 6H and I; Supporting Information Fig. S9A and S9B). Additionally, pretreatment with *α*TLR-4 also reversed the anti-metastatic effects of 3D-PMPs, as indicated by the recovery of bioluminescence signals in metastatic lung tissues of 4T1-Luc tumor-bearing mice both *in vivo* (Fig. 6J) and *ex vivo* (Fig. 6K), as well as H&E staining of lung tissues (Fig. 6L). Collectively, these results highlighted the crucial role of TLR-4 in enabling 3D-PMPs to block platelet-tumor cell interaction, thereby inhibiting tumor metastasis.

### 3.5 Efficient inhibition of tumor growth by DOX@3D-PMPs in orthotopic 4T1-Luc tumor-bearing mice

Following the significant suppression of tumor cell proliferation and metastasis *in vitro*, the antitumor efficacy of DOX@3D-PMPs was evaluated in orthotopic 4T1-Luc breast tumor models. When tumors reached approximately 100 mm^3^, various treatments including PBS, 3D-MPs, PNPs, 3D-PMPs, free DOX, DOX@3D-MPs, DOX@PNPs and DOX@3D-PMPs were administered intravenously to 4T1-Luc tumor-bearing mice at a DOX dosage of 0.5 mg/kg. Doxil, a clinically utilized antitumor drug, served as a positive control at a higher dosage of 4 mg/kg. Expectedly, DOX@3D-PMPs demonstrated the most potent antitumor activity, with 82.8% inhibition in tumor volume (Supporting Information Fig. S10A) and 83.1% inhibition in tumor weight (Fig. S10B) relative to PBS. This exceeded the efficacy of high-dose Doxil, which achieved 63.4% and 57.0% inhibition in tumor volume and weight, respectively, compared to PBS (Fig. S10A and S10B). Kaplan–Meier survival analysis indicated that 37.5% of mice in the DOX@3D-PMPs-treated group were alive 80 days post-treatment, higher than other groups (Fig. S10C). TUNEL staining of tumor sections further confirmed the significant tumor inhibition effects of DOX@3D-PMPs (Supporting Information Fig. S11). No significant change in body weight showed the good biocompatibility of DOX@3D-PMPs (Supporting Information Fig. S12).

### 3.6 Efficient inhibition in tumor metastasis by DOX@3D-PMPs in post-surgical 4T1-Luc breast orthotopic tumor models

**Figure 7:**
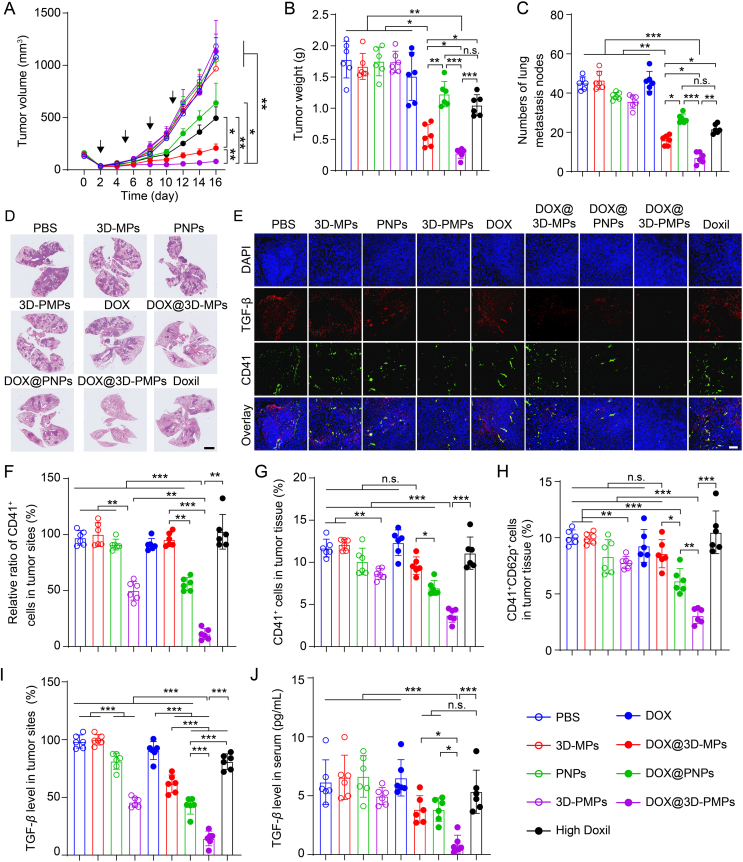
DOX@3D-PMPs markedly enhance inhibition of tumor regrowth and metastasis in a post-surgical orthotopic 4T1-Luc model. (A) Tumor growth curves of post-surgical orthotopic 4T1-Luc tumor-bearing mice following intravenous injection of PBS, 3D-MPs, PNPs, 3D-PMPs, free DOX, DOX@3D-MPs, DOX@PNPs or DOX@3D-PMPs (0.5 mg DOX/kg) or Doxil (4 mg DOX/kg) every three days for four doses, starting 2 days post-surgery. (B) Tumor weight collected at 16 days after treatments. (mean ± SD, *n* = 6). (C) Quantification of lung metastatic nodules at 16 days after treatments indicated in (A). (mean ± SD, *n* = 6). (D) Representative H&E-stained lung sections at 16 days after treatments indicated in (A). Scale bar = 2 mm. (E) Immunofluorescence images of CD41-positive platelets and TGF-*β* level in tumor tissues collected at 16 days after treatments indicated in (A). Scale bar = 50 μm. (F) Quantification of CD41-positive platelets corresponding to (E). (mean ± SD, *n* = 6). (G, H) Flow cytometric analysis of total platelets (G) and activated platelets (H) in tumor tissues from mice treated as in (A). (mean ± SD, *n* = 6). (I) Quantification of TGF-*β* level corresponding to (E). (mean ± SD, *n* = 6). (J) Serum TGF-*β* levels in post-surgical mice at 16 days after treatments indicated in (A). (mean ± SD, *n* = 6). ∗*P* < 0.05, ∗∗*P* < 0.01, ∗∗∗*P* < 0.001 *vs*. indicated; n.s., non-significant (one-way ANOVA followed by Tukey's HSD *post hoc* test for A–C, and F–J).

Given that surgical resection often leads to substantial platelet accumulation at the resection sites, the potential of DOX@3D-PMPs to inhibit post-surgical tumor recurrence was assessed in an orthotopic 4T1-Luc tumor model with incomplete resection. When the tumor volume of 4T1-Luc tumor-bearing mice reached approximately 200 mm^3^, 95% of the tumors were excised, ensuring that the weight of the excised tumor tissues was consistent across the model (Supporting Information Fig. S13). Subsequently, treatments including PBS, 3D-MPs, PNPs, 3D-PMPs, free DOX, DOX@3D-MPs, DOX@PNPs and DOX@3D-PMPs were administered intravenously at a DOX dosage of 0.5 mg/kg every three days for a total of 4 treatments, or Doxil at a higher dosage of 4 mg/kg. As expected, the residual tumors in PBS-, 3D-MPs-, PNPs-, 3D-PMPs- and free DOX-treated groups showed rapid progression (Fig. 7A). While DOX@3D-MPs and DOX@PNPs significantly suppressed tumor recurrence, DOX@3D-PMPs demonstrated the most potent antitumor recurrence capacity, with 92.5% inhibition in tumor volume (Figs. 7A) and 84.3% inhibition in tumor weight (Fig. 7B), better than high-dose Doxil. Compared with controls, DOX@3D-MPs, DOX@PNPs and high-dose Doxil suppressed tumor volume by 80.7%, 40.7% and 54.3%, and reduced tumor weight by 68.4%, 31.1% and 41.1%, respectively (Fig. 7A and B). Furthermore, the capacity of DOX@3D-PMPs to suppress tumor metastasis was evaluated by examining metastatic lung tissues. Analysis of the number of metastatic nodes (Fig. 7C) and H&E staining images (Fig. 7D) revealed minimal lung metastasis in mice treated with DOX@3D-PMPs post-surgery, significantly outperforming DOX@3D-MPs- and high-dose Doxil-treated groups. These results strongly demonstrated the superior ability of DOX@3D-PMPs to inhibit tumor growth and metastasis. The body weight (Supporting Information Fig. S14) and serological analysis (Supporting Information Fig. S15A‒S15F) indicated the relatively good biocompatibility of DOX@3D-PMPs.

To explore the critical role of platelets in the antitumor efficacy of DOX@3D-PMPs, the platelet ratio and activation state within tumor tissues were investigated. Immunofluorescent staining of tumor tissues showed minimal CD41 fluorescence (a specific marker of platelets) in the DOX@3D-PMPs-treated group, only 11.6%, 19.9% and 10.8% of the levels observed in DOX@3D-MPs-, DOX@PNPs- and high-dose Doxil-treated groups, respectively (Fig. 7E and F). Additionally, flow cytometric analysis demonstrated a substantial decrease in the ratios of CD41^+^ cells (Fig. 7G) and CD41^+^CD62p^+^ cells (activated platelets, Fig. 7H) in the DOX@3D-PMPs-treated group, confirming the efficient inhibition of platelet abundance and activation in tumor tissues by DOX@3D-PMPs. Correspondingly, lower levels of TGF-*β*, a key cytokine associated with platelet activation, were observed in tumor tissues (Fig. 7E and I) and serum (Fig. 7J) of the DOX@3D-PMPs-treated group. These findings suggested that DOX@3D-PMPs might significantly suppress post-surgical tumor growth and metastasis by reducing platelet number and their activation.

### 3.7 Robust suppression of lung metastasis by DOX@3D-PMPs in the B16-F10 melanoma metastatic model

To further assess the generalizability of DOX@3D-PMPs, we evaluated their efficacy in a B16-F10 melanoma lung metastasis model. B16-F10 cells were co-injected intravenously with the first treatment dose, followed by two additional administrations every three days across five groups (PBS, DOX@3D-MPs, DOX@PNPs, DOX@3D-PMPs, and Doxil). On Day 25, metastatic burden was assessed by counting lung nodules and performing histological analysis. DOX@3D-PMPs significantly reduced both the number (Supporting Information Fig. S16A and S16B) and size of metastatic nodules (Fig. S16C), outperforming DOX@3D-MPs and high-dose Doxil (4 mg DOX/kg). These results indicated that the therapeutic advantage of 3D-PMPs extends beyond breast cancer to another highly aggressive metastatic model.

### 3.8 Safety evaluation of DOX@3D-PMPs

In addition to evaluating antitumor efficacy, we further assessed the systemic safety of 3D-PMPs, focusing on potential effects on coagulation and immune cell function. As indicated, in mice intravenously injected with 3D-PMPs, tail bleeding time remained unchanged (Supporting Information Fig. S17A), and all coagulation indicators, such as activated partial thromboplastin time (APTT), prothrombin time (PT), thrombin time (TT), and fibrinogen concentration (FIB), were within normal ranges (Fig. S17B‒S17E), indicating that 3D-PMPs do not impair hemostasis or systemic coagulation. We further evaluated potential immunomodulatory effects. Incubation of 3D-PMPs with bone marrow-derived macrophages or primary CD3^+^ T cells did not increase the expression of inflammatory markers (*Tnfa, Cd86,* or *Il6)*, in clear contrast to the activation induced by LPS or PMA controls (Supporting Information Fig. S18A‒S18D). Together, these findings confirm that 3D-PMPs exhibit a favorable safety profile without causing coagulation abnormalities or unintended immune activation.

## 4 Discussion

Platelets exhibit a remarkable capacity to engage in intricate interactions with tumor cells, playing a significant role in promoting tumor development, metastasis and chemoresistance, particularly in post-surgical settings^9^^,^^10^. Thus, the development of comprehensive treatment strategies that not only effectively kill tumor cells but also simultaneously disrupt platelet function to inhibit platelet-driven tumor growth and metastasis is pivotal for enhancing the efficacy of cancer treatments^6^.

Ideal drug delivery systems for tumor cell eradication should be characterized by enhanced tumor accumulation, deep tumor penetration and efficient internalization by tumor cells^35^^,^^36^. The mechanical properties of nanocarriers, particularly their softness, have emerged as a critical factor influencing tumor-targeting efficiency^37^^,^^38^. Our previous work demonstrated that the softness of 3D-MPs significantly enhanced their ability to accumulate in tumors, penetrate deeply and be internalized by tumor cells^28^.

Compared with other membrane-derived drug carriers, platelet membranes offer several distinct advantages^39^. They naturally participate in tumor metastasis and wound-response processes and express adhesion molecules such as P-selectin, enabling direct interaction with circulating tumor cells and preferential localization to metastatic or post-surgical microenvironments^40^, ^41^, ^42^. In addition, platelet membranes lack MHC-I expression and exhibit prolonged circulation, providing inherent immune evasion and enhanced vascular retention, which are not shared by erythrocyte, leukocyte, or cancer-cell membranes^43^. Therefore, we developed a hybrid 3D-PMPs nanocarrier by fusing 3D-MPs with platelet membranes at an optimized protein ratio of 3:1. 3D-PMPs integrated the targeting precision of platelet membranes with the softness of 3D-MPs to enhance drug delivery efficiency. This dual-targeting strategy is essential for overcoming the limitations of conventional drug delivery systems, thereby improving tumor accumulation and penetration, and ensuring efficient cellular uptake and strong cytotoxicity of DOX against tumor cells.

Inhibiting platelet functions has become a promising strategy to combat tumor recurrence and metastasis. Current approaches primarily involve delivering anti-platelet drugs, such as the R300 antibody^18^^,^^19^ and small molecule drugs like aspirin or ticlopidine^16^^,^^17^ to tumor tissues. However, platelets are indispensable for coagulation and vascular integrity, and systemic inhibition or elimination of platelets poses significant bleeding risks, especially in elderly patients or those with coagulation disorders^6^. Platelet decoys have recently emerged as a novel biomimetic strategy for disrupting platelet-mediated tumor metastasis^20^^,^^21^. By retaining platelet membrane structures while eliminating their activation and aggregation capabilities, these decoys mimic platelet functions to competitively block interaction between platelets and tumor cells. Notably, platelet decoys have a reversible inhibitory effect, making them safer compared to traditional anti-platelet drugs, as their effects can be rapidly neutralized by introducing fresh platelets^22^. This feature is particularly advantageous in scenarios requiring intact coagulation, such as post-surgery or trauma. However, the clinical potential of platelet decoys remains limited by their inability to efficiently target tumor tissues. Without effective delivery to the tumor microenvironment, their ability to block platelet-tumor cell interaction is significantly compromised, restricting their therapeutic efficacy. Here, 3D-PMPs had the ability to target tumor tissues and functioned as platelet decoys to inhibit platelet-tumor cell interaction, as shown by the decreased platelet-induced tumor cell proliferation, migration and metastasis both *in vitro* and *in vivo*. Thus, besides directly killing tumor cells, DOX@3D-PMPs effectively inhibited platelet-induced tumor growth and metastasis, exerting strong anticancer activity. Mechanistically, TLR-4 was involved in 3D-PMPs-mediated inhibition in platelet-tumor cell interaction, as evidenced by the fact that pretreatment with *α*TLR-4 significantly promoted platelet-tumor cell interaction and abrogated 3D-PMPs-induced tumor cell proliferation and metastasis.

However, several limitations should be noted. Platelet-derived materials face challenges in large-scale preparation, batch consistency, and donor-dependent activation status, requiring stricter quality control than erythrocyte membranes, which are abundant and easy to isolate^44^. In addition, because platelets participate in thrombosis and inflammation, platelet-based carriers require careful safety evaluation to exclude unintended interactions with coagulation or immune pathways. Furthermore, although the enhanced blocking efficiency of 3D-PMPs is linked to their mechanical softness, the underlying mechanisms governing softness-dependent tumor receptor masking remain unclear. Elucidating this relationship may guide the rational design of soft EV-based systems to disrupt tumor-microenvironment interactions and improve combination cancer therapies.

## 5 Conclusions

In summary, this study shows that DOX@3D-PMPs not only efficiently target tumor tissues to kill tumor cells but also function as platelet decoys to inhibit platelet-triggered tumor growth and metastasis. Our work demonstrates the potential of 3D-PMPs as a novel therapeutic platform to enhance cancer treatment efficacy.

## Acknowledgments

This work was supported by 10.13039/501100012166National Key R&D Program of China (2021YFA1201200, 2022YFA1206000, 2020YFA0710700), 10.13039/501100001809National Natural Science Foundation of China (32571622, 82573379, 32571552, 82272844, 82073796), Program for HUST Academic Frontier Youth Team (2018QYTD01, China), the Fundamental Research Funds for the Central Universities (HUST, 2025BRB002, China). We thank the Research Core Facilities for Life Science (HUST, China), Optical Bioimaging Core Facility of WNLO-HUST and the Analytical and Testing Center of Huazhong University of Science and Technology for related analysis (China).

## Author contributions

Nana Bie: Writing-Original Draft, Investigation, Methodology, Data curation. Shiyu Li: Investigation, Methodology, Data curation. Kaili Sun, Jianye Li, Xin Li, Xiaojuan Zhang: Investigation, Methodology. Muzi Tian, Zixiang Xie, Yixi Xiao, Yujie Zhang, Zixi Wang, Yizhou Huang, Yinmei Zhu: Methodology. Xiangliang Yang, Lu Gan: Conceptualization, Writing-Review & Editing, Funding acquisition. Tuying Yong: Conceptualization, Writing-Review & Editing, Funding acquisition, Data Curation.

## Conflicts of interest

The authors declare no conflicts of interest.

## Supporting information

The following is the Supporting Information to this article.
