# Supplementary material for: Soft cell-derived hybrid microparticles with platelet decoys for enhanced cancer chemotherapy
Source: Acta Pharm Sin B. 2026 Mar 19;16(6):3846–60. doi: 10.1016/j.apsb.2026.03.028 (PMC13304818; doi:10.1016/j.apsb.2026.03.028)
Supplement: Multimedia component 1 [file mmc1.pdf]

Supporting Information for

ORIGINAL ARTICLE

Soft cell-derived hybrid microparticles with platelet decoys for enhanced cancer chemotherapy

Nana Bie[a,†], Shiyu Li[a,†], Kaili Sun[a], Jianye Li[a], Xin Li[a], Xiaojuan Zhang[a], Muzi Tian[a], Zixiang Xie[a], Yixi Xiao[a], Yujie Zhang[a], Zixi Wang[a], Yizhou Huang[a], Yinmei Zhu[a], Xiangliang Yang[a,b,c], Lu Gan[a,b,c], Tuying Yong[a,b,c,*]

[a]*National Engineering Research Center for Nanomedicine, College of Life Science and Technology, Huazhong University of Science and Technology, Wuhan 430074, China*

[b]*Key Laboratory of Molecular Biophysics of the Ministry of Education, College of Life Science and Technology, Huazhong University of Science and Technology, Wuhan 430074, China*

[c]*Hubei Key Laboratory of Bioinorganic Chemistry and Materia Medica, Huazhong University of Science and Technology, Wuhan 430074, China*

Received 15 August 2025; received in revised form 28 November 2025; accepted 7 December 2025

*Corresponding authors.

E-mail address: yongty2018@hust.edu.cn (Tuying Yong)

[†]These authors made equal contributions to this work.

1. Supporting experimental section

1.1. Intracellular Trafficking

4T1 cells were treated with DiO-labelled DOX@3D-PMPs at a DOX concentration of 1 μg/mL at 37 °C for different time intervals. Following three washes with PBS, the cell membrane, lysosomes, or nuclei of the cells were stained with CellVue Claret (100 nmol/L, Sigma–Aldrich, St Louis, USA), LysoTracker Deep Red (75 nmol/L, Yeason, Shanghai, China), or DAPI (5 μg/mL, Beyotime, Shanghai, China), separately. The cells were washed with PBS and detected by confocal laser scanning microscopy (FV3000, Olympus, Japan).

1.2. Impact of 3D-PMPs on BMDMs and T cells

To assess the effect of 3D-PMPs on repolarization of bone marrow-derived macrophages (BMDMs), bone marrow cells were obtained from the femurs and tibias of 6-week-old male BALB/c mice and differentiated into BMDMs by culturing for 5 days in complete RPMI 1640 medium supplemented with 20 ng/mL recombinant mouse macrophage colony-stimulating factor. The BMDMs were treated with 3D-PMPs at a protein concentration of 10 μg/mL or 100 ng/mL LPS serving as the positive control. To assess the effect of 3D-PMPs on T-cell activation, $CD3^+$ T cells were isolated from splenocytes of 6-week-old male BALB/c mice using the MojoSort Mouse CD3 T Cell Isolation Kit (BioLegend, San Diego, CA, USA) and cultured in medium supplemented with 20 ng/mL IL-2. The isolated T cells were then treated with 3D-PMPs at a protein concentration of 10 μg/mL for 24 h, with 25 ng/mL phorbol 12-myristate 13-acetate (PMA) used as the positive control. The cells were harvested and repolarization markers of BMDMs (*Cd86* and *Tnfa*) or activation markers of T cells (*Il6* and *Tnfa*) were detected by real-time reverse transcription-polymerase chain reactions (RT-qPCR). The used primer sequences are as follows: mouse *Gadph* (F: 5′-GTTCCTACCCCCAATGTGTCC-3′, R: 5′-TAGCCCAAGATGCCCTTCAGT-3′); mouse *Tnfa* (F: 5′-CCCACGTCGTAGCAAACCAC-3′, R: 5′-GCAGCCTTGTCCCTTGAAGA-3′); mouse *Cd86* (F: 5′-TTGTGTGTGTTCTGGAAACGGAG-3′, R: 5′-

AACTTAGAGGCTGTGTTGCTGGG-3′); mouse *Il6* (F: 5′-GCCTTCTTGGGACTGATGCT-3′, R: 5′- TGTGACTCCAGCTTATCTCTTGG-3′).

1.3. Biodistribution analysis

When the tumor volume of 4T1 tumor-bearing mice reached approximately 200 mm^3, the mice were intravenously administered with IR780-labeled 3D-PMPs at a dosage of 4 mg protein/kg. At predetermined time points post-administration, the mice were euthanized and the hearts, livers, spleens, lungs, and kidneys were harvested. The NIRF images and average radiant efficiency of organs were acquired using a Caliper IVIS Lumina II *in vivo* imaging system (PerkinElmer, Waltham, MA, USA).

1.4. Impact of 3D-PMPs on in vivo coagulation function

Six-week-old male BALB/c mice were intravenously injected with 3D-PMPs at a dosage of 4 mg protein/kg. At 12 h after administration, tails were transected and immediately immersed in 37 °C saline to assess bleeding time. Subsequently, mouse plasma was collected and isolated using sodium citrate anticoagulant tubes, and activated partial thromboplastin time (APTT), prothrombin time (PT), thrombin time (TT), and fibrinogen concentration (FIB) were measured within 4 hours of collection.

2. Supporting figures

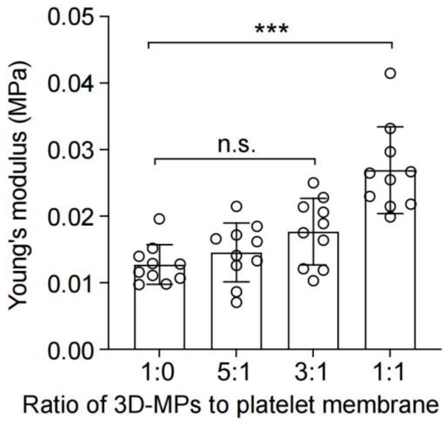

Figure S1 Young's modulus of 3D-PMPs at different ratios of 3D-MPs to fragmented platelet membranes by AFM. Data are presented as mean ± SD (n = 10). ***$P < 0.001$ *vs*. indicated; n.s., non-significant (one-way ANOVA followed by Tukey's HSD *post hoc* test).

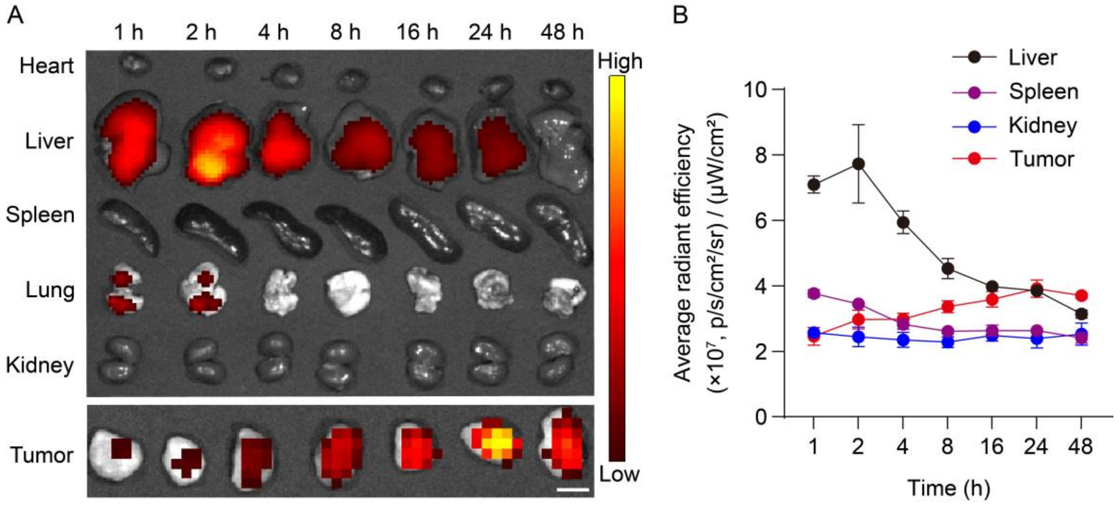

Figure S2 Biodistribution of 3D-PMPs. (A, B) Representative images (A) and quantification (B) of the biodistribution of DiR-labled 3D-PMPs in the major organs, including liver, spleen, kidney and tumor, at the indicated time points. Scale bar = 5 mm. (mean ± SD, *n* = 3)

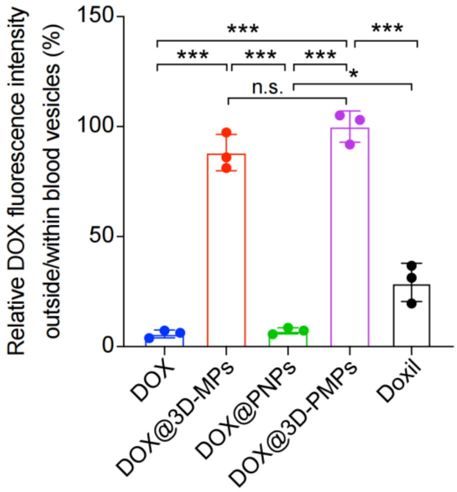

Figure S3 Quantitative analysis of relative DOX fluorescence intensity outside tumor blood vessels in orthotopic 4T1 tumor-bearing mice at 24 h after intravenous injection of free DOX, DOX@3D-MPs, DOX@PNPs or DOX@3D-PMPs at a DOX dosage of 0.5 mg/kg or Doxil at a higher dosage of 4 mg/kg, respectively. Tumor vessels were labeled with FITC-conjugated anti-CD31 antibody. (mean ± SD, n = 3). *$P < 0.05$, ***$P < 0.001$ *vs*. indicated; n.s., non-significant (one-way ANOVA followed by Tukey's HSD *post hoc* test).

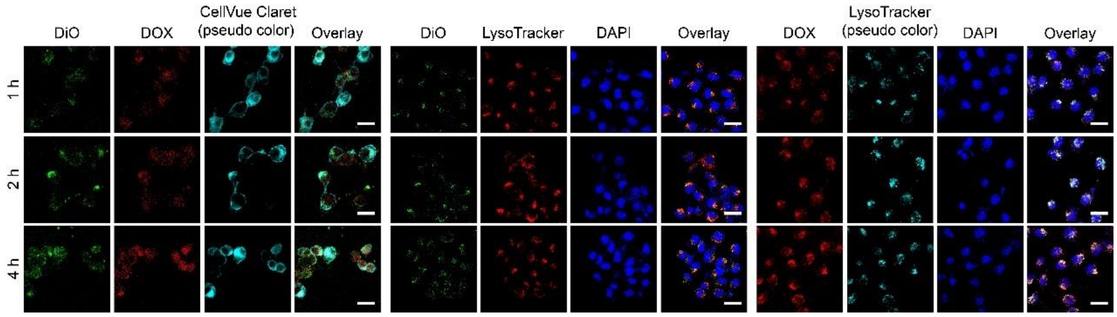

Figure S4 Confocal microscopic images of the intracellular trafficking of DOX-packaging DiO-labeled 3D-PMPs after 4T1 cells were treated with DOX@3D-PMPs at a DOX concentration of 1 μg/mL for different time intervals and then labeled with CellVue Claret (cell membrane labeling dye), LysoTracker Deep Red (lysosomes labeling dye) or DAPI (nucleus labeling dye), respectively. Scale bar = 25 μm.

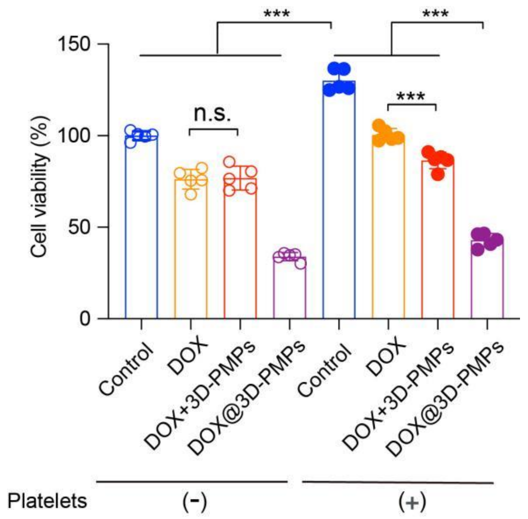

Figure S5 Cell viability of 4T1 cells after treatment with free DOX, the mixture of DOX and 3D-PMPs or DOX@3D-PMPs at a DOX concentration of 1 μg/mL with or without platelets for 24 h. (mean ± SD, $n = 5$). ***$P < 0.001$ *vs*. indicated; n.s., non-significant (one-way ANOVA followed by Tukey's HSD *post hoc* test).

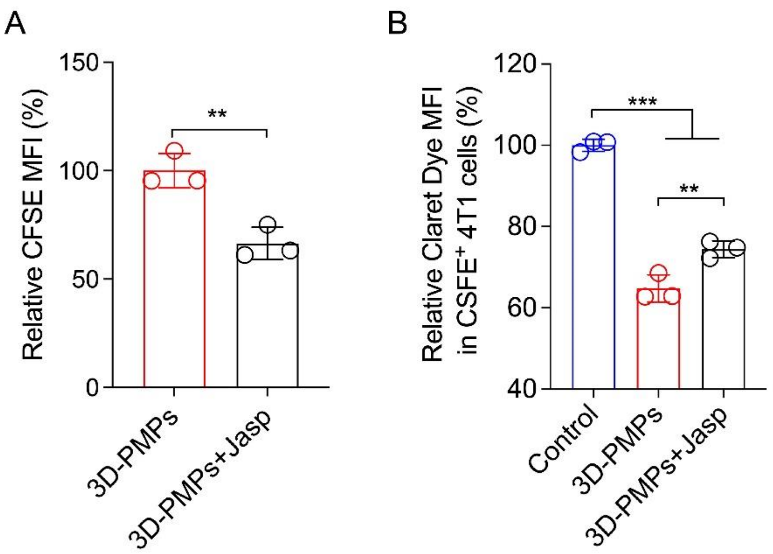

Figure S6 The effect of 3D-PMPs' softness on uptake by 4T1 cells and inhibition of platelet adhesion. (A) Relative CFSE MFI in 4T1 cells after treatment with CFSE-labeled 3D-PMPs, which were pretreated with or without 2 h incubation with 50 nmol/L Jasp, for 4 h. (mean ± SD, *n* = 3). (B) Relative Claret Dye MFI in 4T1 cells after treatment with 3D-PMPs or 3D-PMPs+Jasp for 4 h, followed by incubation with Claret Dye-labeled platelets for 4 h. (mean ± SD, *n* = 3). **P < 0.01, ***P < 0.001 *vs*. indicated (one-way ANOVA followed by Tukey's HSD *post hoc* test).

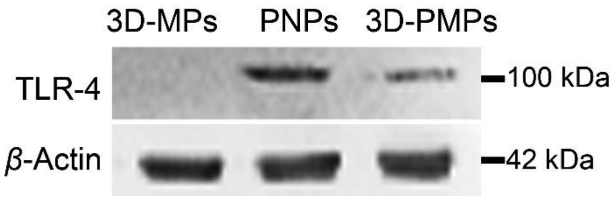

Figure S7 TLR-4 expression on 3D-MPs, PNPs and 3D-PMPs by Western blotting analysis.

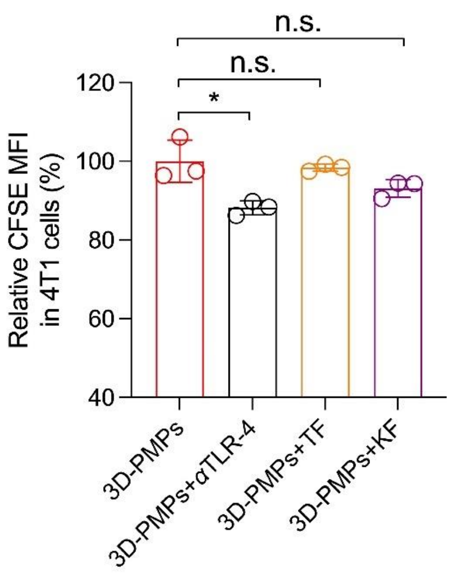

Figure S8 Relative CFSE MFI in 4T1 cells after treatment with CFSE-labeled 3D-PMPs, which were pretreated with or without 2 h incubation with 2 μg/mL *α*TLR-4, 20 nmol/L tirofiban (TF) or 5 μmol/L KF38789 (KF) for 4 h. (mean ± SD, n = 3). *P < 0.01 *vs*. indicated; n.s., non-significant (one-way ANOVA followed by Tukey's HSD *post hoc* test).

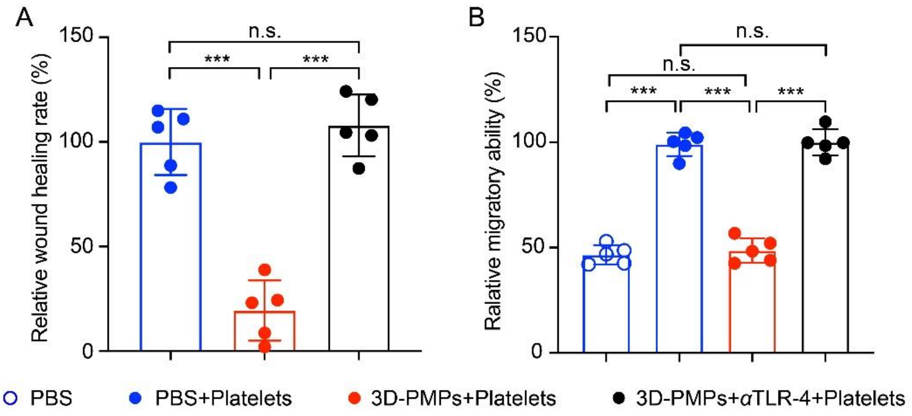

Figure S9 TLR-4 involvement in the 3D-PMPs-triggered inhibition of tumor cell migration. (A, B) Quantitative analysis of wound healing rate (A) and migratory ability (B) of 4T1 cells pre-treated with PBS, 3D-PMPs, or *α*TLR-4-incubating 3D-PMPs at a protein concentration of 10 μg/mL for 4 h, followed by incubation with or without platelets for 18 h. (mean ± SD, $n = 5$). ***$P < 0.001$ *vs*. indicated; n.s., non-significant (one-way ANOVA followed by Tukey's HSD *post hoc* test).

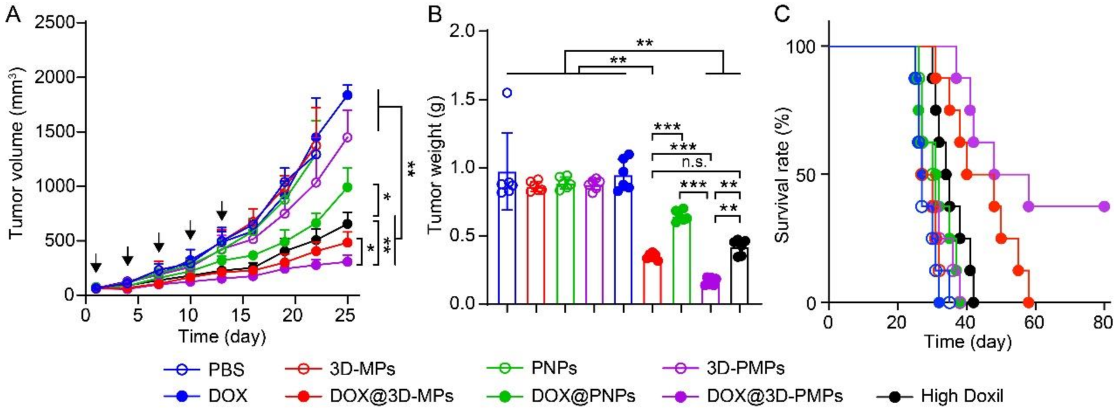

Figure S10 Enhanced tumor inhibition of orthotopic 4T1-Luc tumor-bearing mice by DOX@3D-PMPs. (A) Tumor growth curves of orthotopic 4T1-Luc tumor-bearing mice following intravenous injection of PBS, 3D-MPs, PNPs, 3D-PMPs, free DOX, DOX@3D-MPs, DOX@PNPs or DOX@3D-PMPs at a DOX dosage of 0.5 mg/kg, or high-dose Doxil at 4 mg/kg every three days for 5 times. (mean ± SD, *n* = 6). (B) Weight of tumor tissues at 25 days after treatments indicated in (A). (mean ± SD, *n* = 6). (C) Kaplan–Meier survival plots of 4T1-Luc tumor-bearing mice after treatments indicated in (A). (mean ± SD, n = 8). *$P < 0.05$, **$P < 0.01$, ***$P < 0.001$ *vs*. indicated; n.s., non-significant (one-way ANOVA followed by Tukey's HSD *post hoc* test for A and B).

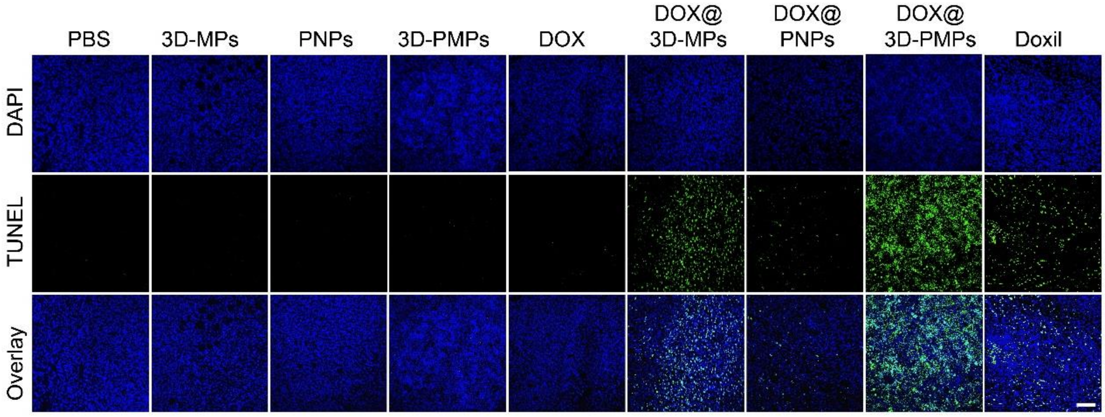

Figure S11 Representative TUNEL staining images of tumor tissues in orthotopic 4T1 tumor-bearing mice at 25 days after intravenous injection of PBS, 3D-MPs, PNPs, 3D-PMPs, free DOX, DOX@3D-MPs, DOX@PNPs or DOX@3D-PMPs at a DOX dosage of 0.5 mg/kg, or Doxil at a higher dosage of 4 mg/kg every three days for 5 times. Scale bar = 50 μm.

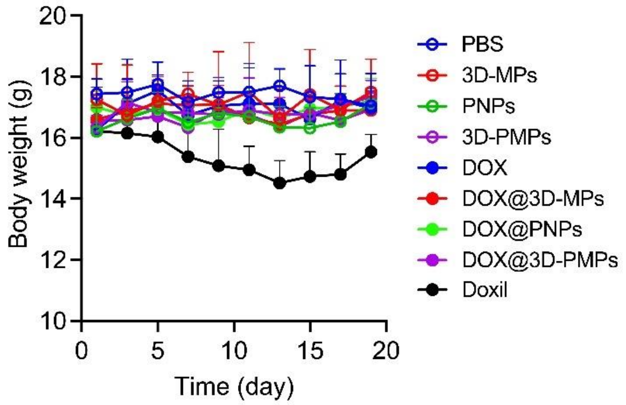

Figure S12 Body weight of orthotopic 4T1-Luc tumor-bearing mice after intravenous injection of PBS, 3D-MPs, PNPs, 3D-PMPs, free DOX, DOX@3D-MPs, DOX@PNPs or DOX@3D-PMPs at a DOX dosage of 0.5 mg/kg, or Doxil at a higher dosage of 4 mg/kg every three days for 5 times. (mean ± SD, $n = 6$).

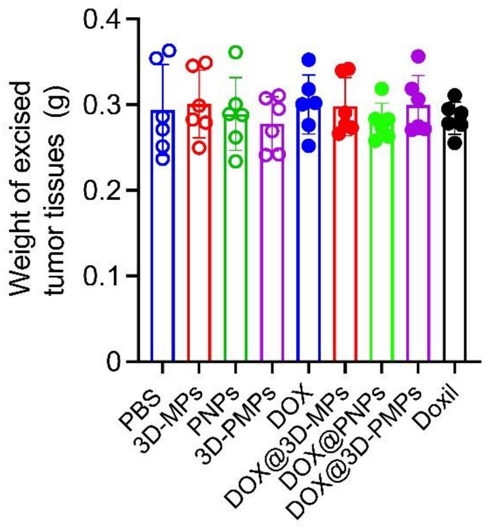

Figure S13 Weight of the excised tumor tissues when tumor volume in orthotopic 4T1-Luc tumor-bearing mice reached approximately 200 mm^3. (mean ± SD, n = 6).

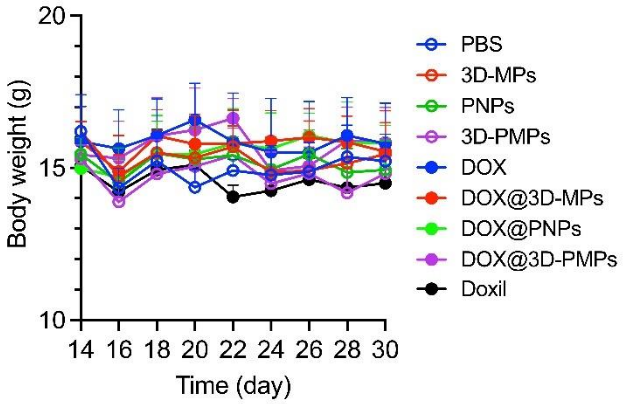

Figure S14 Body weight of post-surgery orthotopic 4T1 tumor-bearing mice after intravenous injection of PBS, 3D-MPs, PNPs, 3D-PMPs, free DOX, DOX@3D-MPs, DOX@PNPs, DOX@3D-PMPs at a DOX dosage of 0.5 mg/kg or Doxil at a higher dosage of 4 mg/kg. (mean ± SD, n = 6).

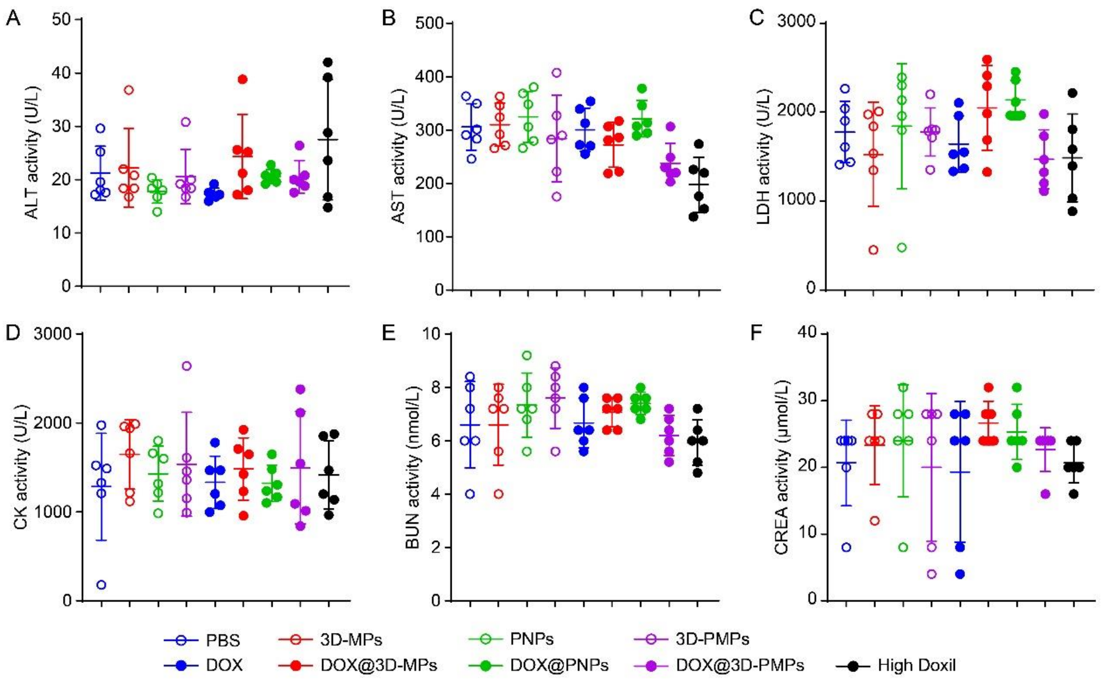

Figure S15 Serological analysis of post-surgery orthotopic 4T1 tumor-bearing mice after intravenous injection of PBS, 3D-MPs, PNPs, 3D-PMPs, free DOX, DOX@3D-MPs, DOX@PNPs, DOX@3D-PMPs at a DOX dosage of 0.5 mg/kg or Doxil at a higher DOX dosage of 4 mg kg. Alanine aminotransferase (ALT, A), aspartate aminotransferase (AST, B), lactate dehydrogenase (LDH, C), creatine kinase (CK, D), blood urea nitrogen (BUN, E) and creatinine (CREA, F) were analyzed at 14 days post-surgery. (mean ± SD, *n* = 6).

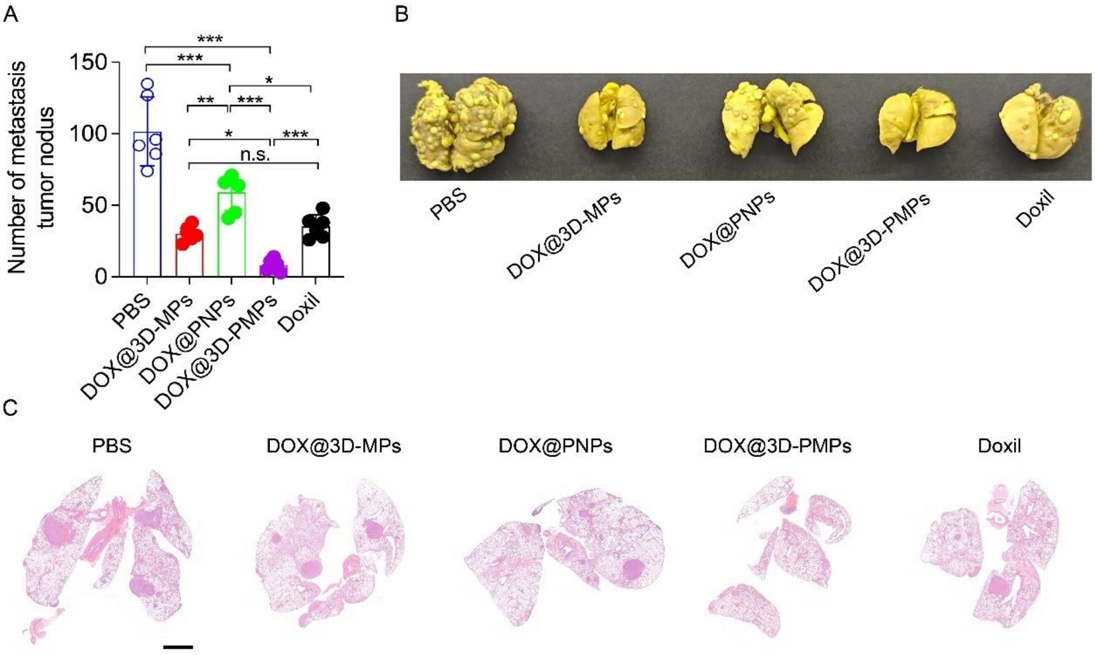

Figure S16 Enhanced tumor inhibition of metastatic B16-F10 tumor-bearing mice by DOX@3D-PMPs. (A) Number of metastasis nodes of lung tissues at 25 days after intravenous injection of PBS, DOX@3D-MPs, DOX@PNPs or DOX@3D-PMPs at a DOX dosage of 0.5 mg/kg, or Doxil at a higher dosage of 4 mg/kg every three days for 3 times. (mean ± SD, $n = 6$). (B) Representative lung tissues at 25 days after treatments indicated in (A). (C) H&E staining images of lung tissues at 25 days after treatments indicated in (A). Scale bar = 2 mm. *$P < 0.05$, **$P < 0.01$, ***$P < 0.001$ *vs*. indicated; n.s., non-significant (one-way ANOVA followed by Tukey's HSD *post hoc* test).

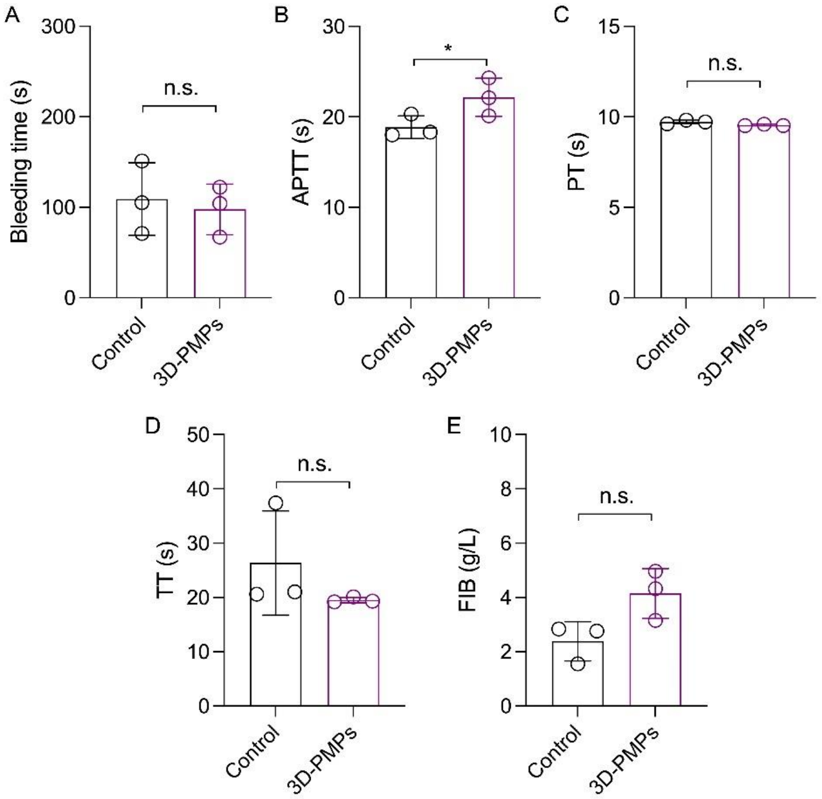

Figure S17 The coagulation function of 3D-PMPs. (A) Bleeding time of mice post-injection with PBS or 3D-PMPs for 12 h. (B-E) Activated partial thromboplastin time (APTT, B), prothrombin time (PT, C), thrombin time (TT, D), and fibrinogen concentration (FIB, E) of plasma from mice post-injection with PBS or 3D-PMPs for 12h. (mean ± SD, n = 3). *$P < 0.05$ *vs*. indicated; n.s., non-significant (two-tailed Student's t-test).

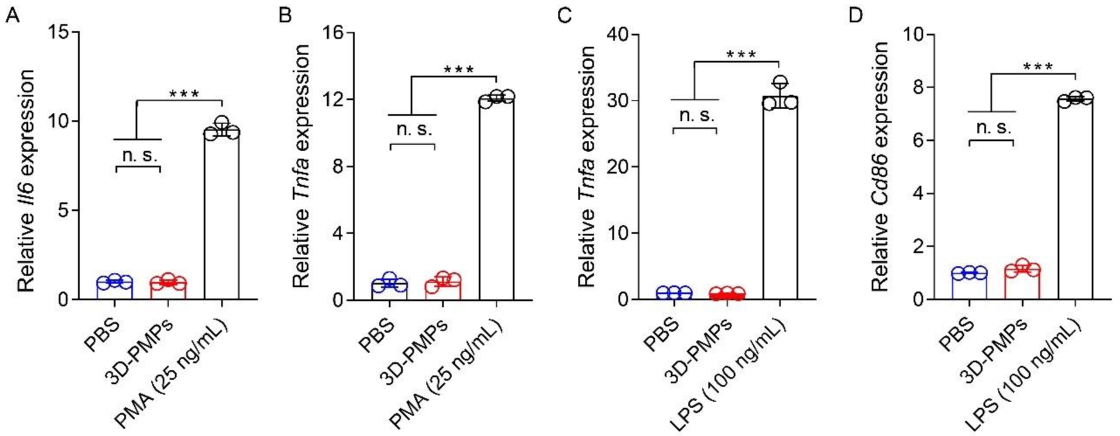

Figure S18 Immunomodulatory assessment of 3D-PMPs by RT-qPCR. (A, B) Relative expression levels of *Il6* (A) and *Tnfa* (B) in $CD3^+$ T cells after treatment with PBS, 3D-PMPs or PMA (positive control). (C, D) Relative expression levels of *Tnfa* (C) and *Cd86* (D) in BMDM cells after treatment with PBS, 3D-PMPs or LPS (positive control). (mean ± SD, $n = 3$). $^{***}P < 0.001$ *vs*. indicated; n.s., non-significant (one-way ANOVA followed by Tukey's HSD *post hoc* test).
